# SIRT3 Modulates Endothelial Mitochondrial Redox State during Insulin Resistance

**DOI:** 10.3390/antiox11081611

**Published:** 2022-08-19

**Authors:** Elisa Martino, Anna Balestrieri, Camilla Anastasio, Martina Maione, Luigi Mele, Domenico Cautela, Giuseppe Campanile, Maria Luisa Balestrieri, Nunzia D’Onofrio

**Affiliations:** 1 Department of Precision Medicine, University of Campania Luigi Vanvitelli, Via L. De Crecchio 7, 80138 Naples, Italy; 2 Food Safety Department, Istituto Zooprofilattico Sperimentale del Mezzogiorno, Via Salute, 2, 80055 Portici, Italy; 3 Department of Experimental Medicine, University of Campania Luigi Vanvitelli, Via Luciano Armanni 5, 80138 Naples, Italy; 4 Stazione Sperimentale per le Industrie delle Essenze e dei Derivati dagli Agrumi (SSEA)—Azienda Speciale CCIAA di Reggio Calabria, 89125 Reggio Calabria, Italy; 5 Department of Veterinary Medicine and Animal Production, University of Naples Federico II, Via F. Delpino 1, 80137 Naples, Italy

**Keywords:** SIRT3, mitochondria, insulin resistance, endothelial cells, δ-valerobetaine

## Abstract

Emerging evidence indicates that defects in sirtuin signaling contribute to impaired glucose and lipid metabolism, resulting in insulin resistance (IR) and endothelial dysfunction. Here, we examined the effects of palmitic acid (PA) treatment on mitochondrial sirtuins (SIRT2, SIRT3, SIRT4, and SIRT5) and oxidative homeostasis in human endothelial cells (TeloHAEC). Results showed that treatment for 48 h with PA (0.5 mM) impaired cell viability, induced loss of insulin signaling, imbalanced the oxidative status (*p* < 0.001), and caused negative modulation of sirtuin protein and mRNA expression, with a predominant effect on SIRT3 (*p* < 0.001). Restoration of SIRT3 levels by mimic transfection (SIRT3^+^) suppressed the PA-induced autophagy (mimic NC+PA) (*p* < 0.01), inflammation, and pyroptosis (*p* < 0.01) mediated by the NLRP3/caspase-1 axis. Moreover, the unbalanced endothelial redox state induced by PA was counteracted by the antioxidant δ-valerobetaine (δVB), which was able to upregulate protein and mRNA expression of sirtuins, reduce reactive oxygen species (ROS) accumulation, and decrease cell death. Overall, results support the central role of SIRT3 in maintaining the endothelial redox homeostasis under IR and unveil the potential of the antioxidant δVB in enhancing the defense against IR-related injuries.

## 1. Introduction

Mitochondria are crucial players in the maintenance of endothelial function and regulation of cellular mechanisms responsible for the onset of inflammation and cell death. Mitochondrial dysfunction occurring in the high-fat condition impairs endothelial homeostasis thus constituting a major risk factor for cardiovascular diseases, including diabetes, heart failure, pulmonary hypertension, and insulin resistance (IR) [1]. In this contest, mitochondrial sirtuins (SIRT) contribute to cellular homeostasis due to their pivotal roles in controlling metabolic processes as well as the responses to nutrient availability and oxidative stress [1,2,3]. SIRT, class III histone deacetylases, represent attractive tools for epigenetic intervention due to their crucial role in DNA damage repair and control of cell survival and metabolic homeostasis, including IR [3,4,5].

IR refers to a condition in which insulin-responsive cells undergo a reduced response to insulin, caused by disruption of specific events in the signaling pathways resulting in the onset of diseases such as type 2 diabetes [6]. At the vascular level, insulin signaling begins with the binding of insulin to its receptor, a tyrosine kinase which undergoes autophosphorylation of intracellular tyrosine residues, resulting in the recruitment and phosphorylation of target proteins, such as insulin receptor substrates IRS1 and IRS2 [7]. The activation of IRS1 determines the phosphorylation of several downstream target proteins, such as protein kinase B (Akt), culminating in endothelial IR impairment of IRS1/PI3K/Akt/eNOS signaling and leading to a series of systemic metabolic abnormalities [8,9,10]. In the state of sustained hyperglycemia occurring under IR, endothelial reactive oxygen species (ROS) markedly increase, resulting but not limited to oxidative stress and chronic inflammation, which even cause apoptosis, pyroptosis, and necrosis. Pyroptosis, or caspase-1-dependent cell death, is a typical proinflammatory programmed endothelial cell death related to the development of obesity-associated inflammation, diabetes, and IR [11,12,13,14,15].

In the contest of emerging epigenetic modulators, several studies have demonstrated the effects of the natural antioxidant δ-valerobetaine (δVB), *N*,*N*,*N*-trimethyl-5-aminovaleric acid [16,17,18,19,20,21,22,23], a water-soluble trimethylated compound originating from a specific transformation of Nε-trimethyllysine [24,25]. Experimental studies suggest that δVB has great potential in the prevention of diabetes and its vascular complications, with many effects occurring through the modulation of the epigenetic regulators, SIRT1 and SIRT6 [16,17,18,19]. However, the effects of δVB on mitochondrial homeostasis in endothelial cells have not yet been described. Here, the oxidative stress elicited by increased intracellular levels of ROS and free fatty acids occurring under PA treatment was used to investigate the role of mitochondrial SIRT2, SIRT3, SIRT4, and SIRT5 on endothelial redox homeostasis function. Moreover, we tested the potential of δVB against PA-induced mitochondrial dysfunction.

## 2. Materials and Methods

### 2.1. Cell Culture and Treatments

Endothelial cells (EC) from human aorta (TeloHAEC cell line, CRL-4052) were obtained from the American Type Culture Collection (ATCC, Manassas, VA, USA) and grown as a monolayer, at 37 °C under a humidified atmosphere with 5% CO_2_, in Vascular Cell Basal Medium (PCS-100-030, ATCC, Manassas, VA, USA) supplemented with Endothelial Cell Growth kit-VEGF (PCS-100-041, ATCC, Manassas, VA, USA). To induce insulin-resistance (IR), EC were exposed to palmitic acid (PA) up to 1.5 mM for 24 and 48 h. To confirm the IR assessment, the 48 h treatment with PA was followed by incubation with 100 nM recombinant human insulin (91077C, Merck KGaA, Darmstadt, Germany) for 30 min, as previously described [26]. δ-Valerobetaine (δVB) synthesis and purification were carried out as previously described [25]. δVB was dissolved in Hanks’ balanced salt solution (HBSS)-10 mM Hepes and treatments were performed for a maximum time of 72 h with betaine concentrations from 0.1 up to 1 mM. For combined treatments (δVB+PA), EC were pre-treated for 16 h with 0.5 mM δVB, followed by removal of δVB and exposure to PA (0.5 mM) for 48 h. Control cells (Ctr) were maintained in a complete culture medium with the corresponding volume of HBSS-10 mM Hepes.

### 2.2. Cell Viability Detection

EC viability was detected using the Cell Counting Kit-8 (CCK-8, Donjindo Molecular Technologies, Inc., Rockville, MD, USA) according to the manufacturer’s instructions. Briefly, CCK-8 solution (10 μL) was added to each well and the plate was incubated at 37 °C for 4 h. Cell absorbance was measured at 450 nm with a microplate reader (model 680, Bio-Rad, Hercules, CA, USA) and viability was expressed as the mean of the optical density at 450 nm. All experiments were performed with *n* = 5 replicates.

### 2.3. SIRT3 Overexpressing

For transient overexpression of SIRT3, EC were seeded in 6-well plates at a density of 1.2 × 10^4^ cells/well. The next day, the 70–80% subconfluent cultures were transfected with 20 nM SIRT3 AAV Vector for specific human Sirtuin 3 (438081010110, Applied Biological Materials, Inc. Richmond, BC, Canada) or miRNA mimic Negative Control (MCH00000, Applied Biological Materials, Inc. Richmond, BC, Canada), in serum- and antibiotic-free medium, using Lullaby (LL70500, OZ Biosciences, Marseille, France) as transfection reagent. EC were incubated for 6 h, followed by an additional 12 h of incubation after the addition of FBS, before treatments. In EC overexpressing SIRT3, mimic Negative Control (mimic NC) was used as a control. The transfection efficiency was confirmed by immunoblotting.

### 2.4. Glucose Uptake Analysis

Intracellular glucose content was determined using the Glucose Assay Kit-WST (G264, Dojindo Molecular Technologies, Inc., Rockville, MD, USA), according to the manufacturer’s protocol. The absorbance of the WST formazan dye was measured at 450 nm by using a microplate reader (model 680, Bio-Rad, Hercules, CA, USA) and the glucose amount in each sample was calculated by plotting absorbance values with the standard calibration curve. Experiments were performed with *n* = 4 replicates.

### 2.5. Lactate Dehydrogenase (LDH) Assay

The integrity of the cell membrane related to LDH release was assessed by the LDH Assay Kit-WST (CK12, Dojindo Molecular Technologies, Inc., Rockville, MD, USA), according to the manufacturer’s instructions. The working solution (100 µL) was added to the cell suspension (50 µL), followed by a 30 min incubation at room temperature in the dark. The absorbance was measured with a microplate reader (model 680, Bio-Rad, Hercules, CA, USA) at 490 nm, and the LDH content, expressed as a percentage of cytotoxicity, was calculated using the following equation: (Test Substance − Low Control)/(High Control − Low Control) × 100. Experiments were performed with *n* = 4 replicates.

### 2.6. Lipid Peroxidation Detection

Lipid peroxidation was determined by measuring the cellular content of malondialdehyde (MDA), according to the manufacturer’s instructions, with the colorimetric lipid peroxidation assay kit (ab118970, Abcam, Cambridge, UK). Following treatment, EC were harvested, homogenized in 303 µL of MDA lysis solution, and 600 µL of thiobarbituric acid (TBA) reagent was added prior to 1 h of incubation at 95 °C and cooling on ice. The absorbance of the supernatant, containing MDA-TBA adduct, was measured at 532 nm with a microplate reader (model 680, Bio-Rad, Hercules, CA, USA). Total MDA levels, normalized to protein content, were calculated by comparing sample absorbance to a standard curve.

### 2.7. Cytokine Levels Determination

Levels of cytokines (IL-6, IL-18, IL-1β, and TNF-α) were measured using ELISA assays (human IL-6 Immunoassay, 6050, R&D Systems, Inc., Minneapolis, MN, USA, Inc.; human interleukin-18 ELISA, RAF143R, BioVendor Laboratorni medicina a.s., Brno, Czech Republic; human IL-1β quantikine ELISA, DLB50, R&D Systems, Inc., Minneapolis, MN, USA, Inc.; and ELISA Cymax TNF-alpha ELISA, YIF-LF-EK0193, AbFrontier, Seoul, Korea, respectively), according to the manufacturer’s protocols. EC lysates were incubated for 1 h in precoated plates with specific anti-cytokine antibodies. After washing to remove unbound cytokines, biotin-labeled antibodies for each cytokine were added and incubated for another hour, followed by a 30 min incubation with streptavidin-HRP conjugate antibodies. The absorbance was measured at 450 nm using a microplate reader (model 680, Bio-Rad, Hercules, CA, USA) and cytokine levels in samples were determined by plotting the absorbance values against the concentrations of each standard curve.

### 2.8. Evaluation of ROS

Intracellular and mitochondrial ROS content was determined with CellROX Green Reagent (C10444, Invitrogen, Waltham, MA, USA) and MitoSOX Red Mitochondrial Superoxide Indicator (M36008, Invitrogen, Waltham, MA, USA), according to manufacturer’s instructions. After treatments, EC were stained for 30 min with 5 µM CellROX or MitoSOX fluorogenic probes in complete medium. Cells were imaged on a fluorescence microscope using the EVOS FL Cell Imaging System (Thermo Scientific, Rockford, IL, USA) and intracellular and mitochondrial ROS fluorescence intensities were quantified using a BD Accuri C6 cytometer (BD Biosciences, San José, CA, USA). At least 20,000 events were recorded for each sample, and analysis was performed with FLOWJO V10 software (Williamson Way, Ashland, OR, USA). Extracellular ROS content was assessed with the Amplex Red Hydrogen Peroxide/Peroxidase Assay kit (A22188, Invitrogen, Waltham, MA, USA). Amplex red reagent (100 μL), containing 50 μM Amplex Red and 0.1 U HRP/ mL, was added to EC suspension (20 μL), containing 2 × 10^4^ live cells in Krebs-Ringer phosphate glucose buffer. After 1 h of incubation at 37 °C, the fluorescence of the oxidized product, 10-acetyl-3,7-dihydroxyphenoxazine, was measured with a multiplate reader (model Infinite 2000, Tecan, Männedorf, Switzerland) using a 530 nm excitation wavelength and a 590 nm emission wavelength. H_2_O_2_ extracellular content was quantified using a standard curve (0–2 μM concentration range). Experiments were performed with *n* = 4 replicates. ROS-inducer menadione (50 µM) (M57405, Sigma Aldrich, St. Louis, MO, USA) was incubated for 30 min at 37 °C and used as a positive control.

### 2.9. Programmed Cell Death Mechanisms

Pyroptosis was detected by using the Pyroptosis/Caspase-1 assay (9145, ImmunoChemistry Technologies, Davis, CA, USA), according to the company’s instructions. After treatments, cells were stained with FLICA caspase-1 reagent (FAM-YVAD-FMK) 1 µL/ mL medium for 1 h in the dark, before fluorescence microscopy with the EVOS FL Cell Imaging System (Thermo Scientific, Rockford, IL, USA). Nigericin (5 µM) was used as a positive control.

Lysosomal acidification was detected by labeling for 30 min at 37 °C in the dark treated EC with 1 µM LysoTracker Red DND-99 (L7528, Invitrogen, Waltham, MA, USA). The autophagic mechanism was assessed with the autophagy assay kit (ab139484, Abcam, Cambridge, UK), following the manufacturer’s instructions. Cells were then washed with PBS and imaged on a fluorescence microscope EVOS FL Cell Imaging System (Thermo Scientific, Rockford, IL, USA) before FACS analysis. Rapamycin (1 µM overnight) was used as a positive control.

Apoptotic mechanisms were investigated with the Annexin V Apoptosis detection kit (556547, BD Pharmigen, Franklin Lakes, NJ, USA), in order to distinguish viable from necrotic and apoptotic cells. After treatments, EC were detached by trypsinization and washed with PBS, and were incubated in 200 μL binding buffer 1× containing 2 μL Annexin V-FITC and 2 μL PI (20 μg/mL) for 30 min.

For all programmed cell mechanisms, flow cytometry analysis was performed using a BD Accuri C6 cytometer (BD Biosciences, San José, CA, USA). For each sample, at least 20,000 events were recorded and the data analyzed by FlowJo V10 software (Williamson Way, Ashland, OR, USA).

### 2.10. Cell Lysis and Immunoblotting Analysis

EC were lysed in buffer (1% NP-40, 0.5% sodium deoxycholate, 0.1% SDS in phosphate buffered saline, PBS) containing 10 μg/mL aprotinin, leupeptin, and 1 mM phenylmethylsulfonylfluoride. Proteins (20–80 μg) were separated by sodium dodecyl sulfate-polyacrylamide gel electrophoresis (SDS-PAGE) and then transferred to nitrocellulose membranes. Non-specific binding sites were blocked for 1 h at room temperature in 1× TBS 1% casein blocker (1610782, Bio-Rad, Hercules, CA, USA). Membranes were then incubated at 4 °C overnight with specific primary antibodies: anti-insulin receptor substrate 1 (phospho S312) (IRS1,1:1000, ab138456, Abcam, Cambridge, UK); anti-phospho-Akt (Thr308) (1:1000, 13038, Cell Signaling Technology, Danvers, MA, USA); anti-Akt (1:2000, 2920, Cell Signaling Technology, Danvers, MA, USA); anti-glycogen synthase kinase-3β (phospho-Ser9) (GSK-3β 1:1000, 9336, Cell Signaling Technology, Danvers, MA, USA); anti-superoxide dismutase 2 (SOD2, 1:2000, sc-137254, Santa Cruz Biotechnology, Dallas, TX, USA); anti-SOD2 (acetyl K68) (1:500, ab137037, Abcam Cambridge, UK); anti-tumor necrosis factor alpha (TNF-α, 1:1000, ab6671, Abcam, Cambridge, UK), anti-nuclear factor kappa B (NF-κB, 1:2000, ab16502, Abcam, Cambridge, UK); anti-interleukin-6 (IL-6, 1:1000, ab229381, Abcam, Cambridge, UK); anti-SIRT2 (1:1000, E-AB-64930-120, Elabscience Biotechnology Inc., Houston, TX, USA); anti-SIRT3 (1:2000, PA5-86035, Invitrogen, Waltham, MA, USA); anti-SIRT4 (1:1000, ab231137, Abcam, Cambridge, UK); anti-SIRT5 (1:1000, ab259967, Abcam, Cambridge, UK); anti-NLR Family Pyrin Domain Containing 3 (NLRP3,1:1000, ab270449, Abcam, Cambridge, UK), anti-caspase-1 (1:500, sc-56036, Santa Cruz Biotechnology, Dallas, TX, USA); anti-IL-1β (1:1000, ab216995, Abcam, Cambridge, UK); anti-IL-18 (1:1000, ab243091, Abcam, Cambridge, UK); anti-target of methylation-induced silencing/apoptosis-associated speck-like protein containing a CARD (TMS1/ASC,1:1000, ab283684, Abcam, Cambridge, UK); anti-autophagy related 5 (ATG5, 1:1000, 9980, Cell Signaling Technology, Danvers, MA, USA); anti-beclin-1 (1:1000, 4122, Cell Signaling Technology, Danvers, MA, USA); anti-sequestosome-1 (SQSTM1/p62, 1:2000, 5114, Cell Signaling Technology, Danvers, MA, USA); anti-microtubule-associated protein 1 light chain 3B (LC3B, 1:2000, ab192890, Abcam, Cambridge, UK); anti-α-tubulin (1:5000, E-AB-20036, Elabscience Biotechnology Inc., Houston, TX, USA); anti-actin (1:3000, ab179467, Abcam, Cambridge, UK), and anti-glyceraldehyde-3-phosphate dehydrogenase (GAPDH, 1:2000, ab9485, Abcam, Cambridge, UK). After 1 h incubation with peroxidase-conjugated secondary antibodies, immunocomplexes were revealed on dried membranes by using the Excellent Chemiluminescent Substrate kit (E-IR-R301, Elabscience Biotechnology Inc., Houston, TX, USA) and acquired by ChemiDoc Imaging System with Image Lab 6.0.1 software (Bio-Rad Laboratories, Milan, Italy). The densities of immunoreactive bands were measured by ImageJ software 1.52n version (Wayne Rasband, National Institutes of Health, Bethesda, MD, USA) and expressed as arbitrary units (AU). In detail, after background subtraction, the density of each band was compared with the loading control signal as well as for each marker involved in protein ratio, as LC3B II/I, phospho-Akt/Akt, and acetyl-SOD2/SOD2.

### 2.11. RNA Isolation and Quantitative RT-PCR

Total RNA was extracted from EC using a Total RNA purification kit (17200, NorgenBiotek Corp., Thorold, ON, Canada), according to the company’s instructions, and then reverse transcribed into cDNA using a Tetro cDNA Synthesis Kit (BIO-65043, Meridian Bioscience Inc., Cincinnati, OH, USA), following the manufacturer′s protocol, on a thermal cycler SureCycler 8800 (Agilent Technologies, Santa Clara, CA, USA). Quantitative real-time assays were carried out using a CFX96 Real-Time PCR Detection System (Bio-Rad, Hercules, CA, USA) with a QuantiTect SYBR Green PCR Kit (204,143, Qiagen, Hilden, Germany) and the following primers (0.2 µM):

− SIRT2 (gene ID: 22933): F-5′-GCCCTTTACCAACATGGCTG-3′, R-5′-TTCGTACAACACCCAGAGCG-3′;
− SIRT2 SIRT3 (gene ID: 23410): F-5′-AGAAGAGATGCGGGACCTTG-3′, R-5′-GGTCCATCAAGCCTAGAGCAG-3′;
− SIRT2 SIRT4 (gene ID: 23409): F-5′-GGCAGGAATCTCCACCGAAT-3′, R-5′-GCACTCCGGACAAAATCACC-3′;
− SIRT2 SIRT5 (gene ID: 23408): F-5′-GGTGTTCCGACCTTCAGAGG-3′, R-5′-GTGGTAGAACTCCCACACCC-3′;
− SIRT2 GAPDH (gene ID: 2597): F-5′-GAAGGTGAAGGTCGGAGTC-3′, R-5′-GAAGATGGTGATGGGATTTC-3′.

The relative SIRT gene expression levels were determined by comparing the expression of each sirtuin to that of GAPDH using the 2^−ΔΔCt^ method [ΔΔ cycle threshold, Ct = (Ct SIRT-Ct GAPDH) of treated EC-(Ct SIRT-Ct GAPDH) of control], and the data was reported as the mean ± SD of *n* = 3 independent experiments, with each reaction performed in triplicate.

### 2.12. Confocal Laser Scanning Microscopy

EC were seeded on microscope glass and fixed for 20 min in a 4% (*v*/*v*) paraformaldehyde solution. Cell permeabilization was performed with 0.1% (*v*/*v*) Triton X-100 in PBS for 10 min. Caspase-1 and NF-κB immunofluorescence was performed by overnight incubation with specific antibodies (1:500) followed by 1 h incubation with Alexa Fluor 633 (1:1000, A21071, Invitrogen, Waltham, MA, USA). For cytoskeleton staining anti-vimentin antibody (1:1000, V6630, Sigma Aldrich, St. Louis, MO, USA) and Alexa Fluor 488 (1:1000, A32723, Invitrogen, Waltham, MA, USA) secondary antibody were used, while nuclear staining was performed for 7 min with 2.5 µg/mL 4′,6-diamidino-2-phenylindole (DAPI; Sigma Aldrich, St. Louis, MO, USA). Immunofluorescence analysis was performed with a confocal microscope (model LSM 700, Zeiss, Oberkochen, Germany) equipped with a plan apochromat X63 (NA1.4) oil immersion objective, and fluorescence intensity, reported as arbitrary fluorescence units (AFU), was estimated with ImageJ 1.52 n software (Wayne Rasband, National Institutes of Health, Bethesda, MD, USA).

### 2.13. Statistical Analysis

All experiments were conducted at least three times, and results were reported as mean ± SD. Statistical analysis between two groups was performed using the Student’s *t* test, while the differences among three groups were analyzed by one-way ANOVA followed by Tukey post hoc test. Analyses and graphs were performed using GraphPad Prism version 9.1.2. Differences with *p* < 0.05 were considered statistically significant.

## 3. Results

### 3.1. Effects of PA on IR, Mitochondria Oxidative Status, and Sirtuins

**Figure 1:**
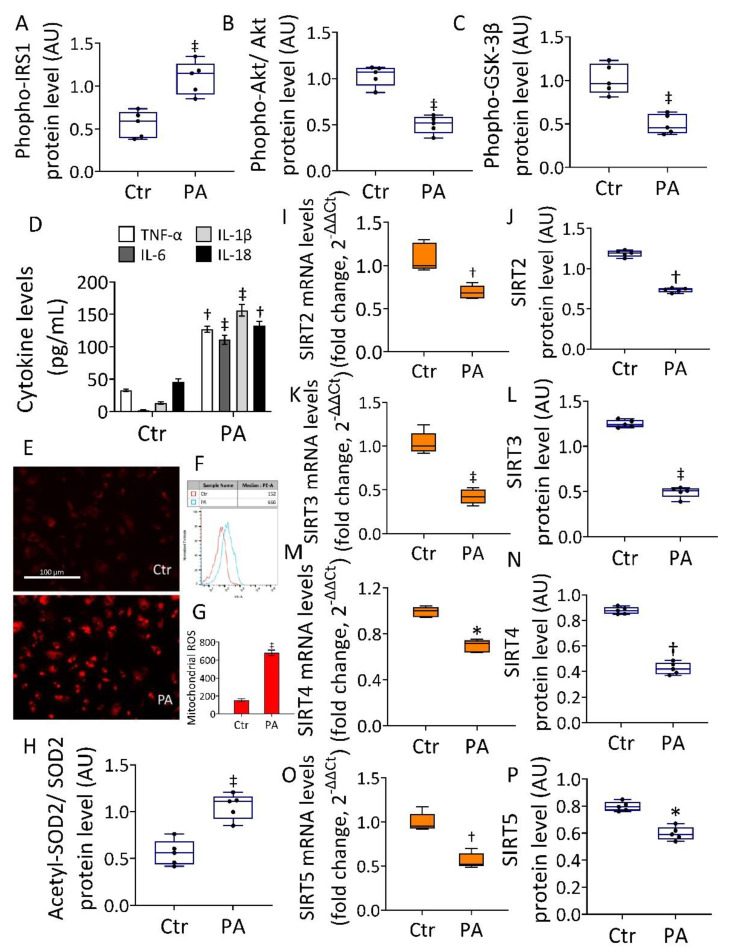
Effects of PA on IR and mitochondrial status. Immunoblotting analysis of (**A**) phospho-IRS1, (**B**) phospho-Akt/Akt, and (**C**) phospho-GSK-3β. (**D**) Cytokine content and (**E**–**G**) representative images by fluorescence microscopy and FACS analysis of mitochondrial ROS detection expressed as fluorescence median ± SD (*n* = 3). Scale bars = 100 µm. (**H**) Acetylated-SOD2/SOD2 ratio. (**I**,**J**) SIRT2, (**K**,**L**) SIRT3, (**M**,**N**) SIRT4, and (**O**,**P**) SIRT5 mRNA and protein levels in EC exposed to 0.5 mM PA for 48 h or treated with the corresponding highest volume of HBSS-10 mM Hepes (Ctr). Western blotting results (*n* = 5) are expressed as arbitrary units (AU), while mRNA levels (*n* = 3) are reported as floating bars with a line representing the median ± SD. * *p* < 0.05 vs. Ctr; † *p* < 0.01 vs. Ctr; ‡ *p* < 0.001 vs. Ctr.

In vitro assays on EC were used as an IR model induced by PA. The results showed the cytotoxic effects induced by PA, with the most relevant effect after treatment with 0.5 mM PA for 48 h (*p* < 0.001) (Figure S1A). The IR was confirmed by decreased glucose uptake (Figure S1B), upregulated phospho-IRS1 protein, reduced phospho-Akt/Akt ratio, and phospho-GSK-3β protein (*p* < 0.001) (Figure 1 and Figure S6).

The IR assessment was also confirmed by the evaluation of phospho-IRS1, phospho-Akt/Akt ratio, and phospho-GSK-3β protein content under PA exposure with or without insulin treatment (Figures S1 and S13). Results indicated that PA+Insulin was unable to revert the PA-induced phospho-IRS1 increase, as well as the decreased phospho-Akt/Akt and phospho-GSK-3β levels (Figures S1 and S13), thus confirming the IR state. Moreover, exposure to PA resulted in an increased cytokine content (*p* < 0.001) and mitochondrial ROS production (Figure 1). Notably, an increased acetylated-SOD2/SOD2 ratio, known to be linked to the suppression of SOD2 activity, was also observed (*p* < 0.001) (Figure 1 and Figure S6). Evaluation of protein and mRNA levels of SIRT2, SIRT3, SIRT4, and SIRT5 showed a decreased expression following PA exposure, with the greatest effect on SIRT3 (0.539 ± 0.03 vs. 1.277 ± 0.04 AU in Ctr, *p* < 0.001) (Figure 1K,L and Figure S6), suggesting the critical involvement of this sirtuin in the redox state of EC during IR.

### 3.2. SIRT3^+^ Decreased the PA-Induced Cytotoxicity

**Figure 2:**
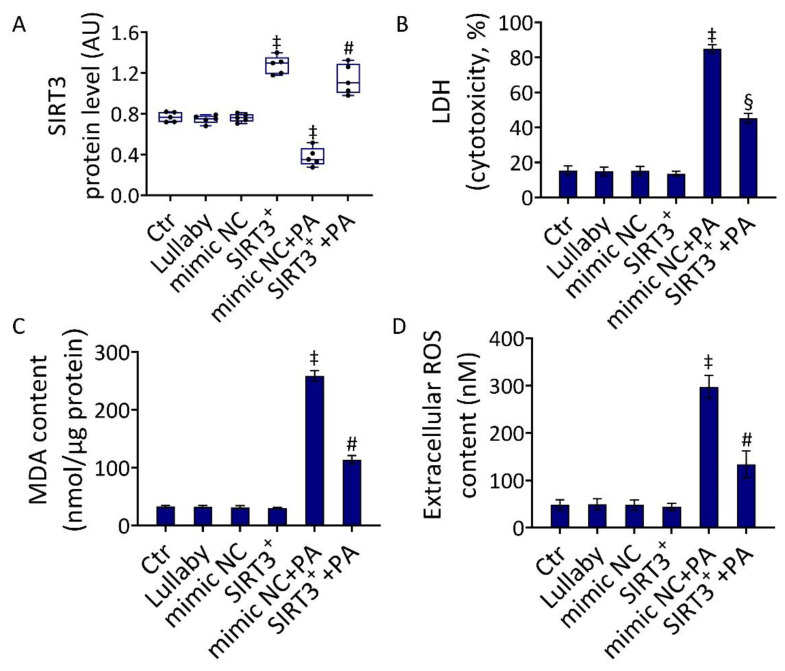
SIRT3^+^ reduced the PA-related cytotoxicity. (**A**) Immunoblotting analysis of SIRT3 protein levels, (**B**) LDH assay cytotoxicity, (**C**) MDA, and (**D**) extracellular ROS content in EC treated with the empty transfection reagent (Lullaby) or transfected with mimic Negative Control (mimic NC), SIRT3 mimic (SIRT3^+^), mimic Negative Control and then exposed to 0.5 mM PA for 48 h (mimic NC+PA) or SIRT3 mimic before 48 h treatment with PA (SIRT3^+^+PA). Control cells (Ctr) were treated with the corresponding volume of HBSS-10 mM Hepes. Western blotting results (*n* = 5) are expressed as arbitrary units (AU) and represented as boxplots. ‡ *p* < 0.001 vs. mimic NC; § *p* < 0.05 vs. mimic NC+PA; # *p* < 0.01 vs. mimic NC+PA.

The evidence that the cytotoxicity of PA was linked to mitochondrial damage and negative modulation of SIRT3, prompted us to investigate the effect of SIRT3 overexpression (SIRT3^+^) on the in vitro IR conditions (Figure 2).

The induction of SIRT3^+^ was confirmed by the evaluation of protein levels (Figure 2A and Figure S7). When the efficacy of SIRT3^+^ to oppose the PA-induced cytotoxicity was investigated (SIRT3^+^+PA), results indicated that SIRT3^+^ inhibited the LDH release (*p* < 0.05 vs. mimic NC+PA), as well as the increase in MDA content (*p* < 0.01 vs. mimic NC+PA) and the accumulation of extracellular ROS (*p* < 0.01 vs. mimic NC+PA) (Figure 2B,D). Based on the evidence that in our experimental conditions, the mimic negative control (mimic NC) showed no cytotoxic effects compared to the control (Ctr) (Figure 2), the mimic NC was used as a control for further experiments.

### 3.3. SIRT3^+^ Reverted the PA-Induced IR State and Oxidative Stress

**Figure 3:**
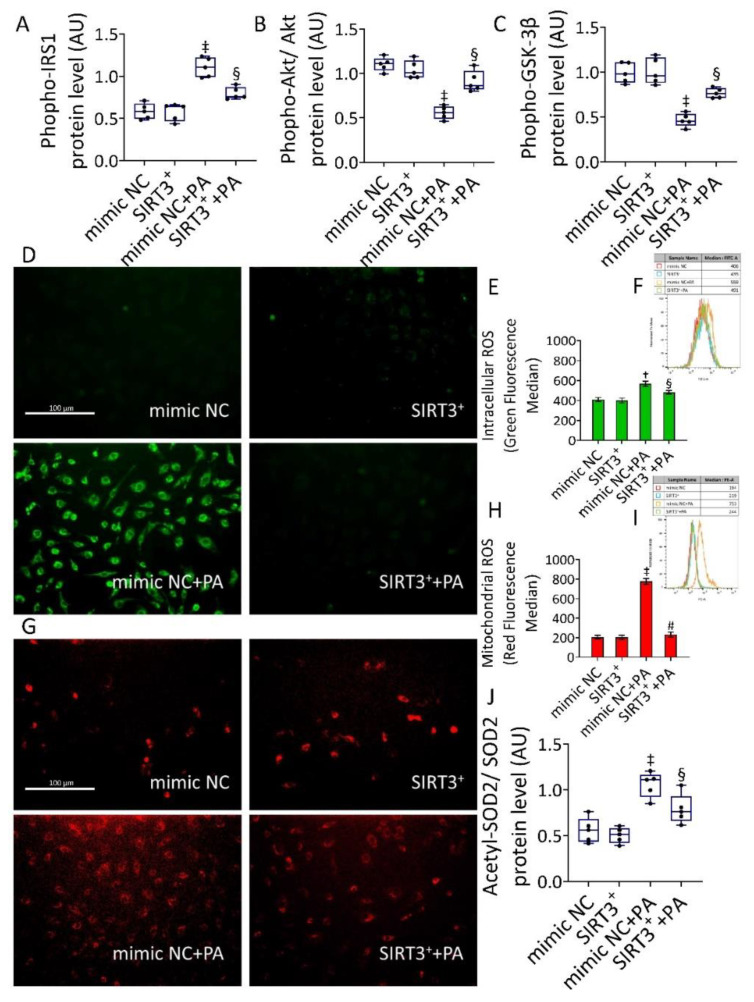
SIRT3^+^ decreased the PA-induced IR and oxidative stress. (**A**) phospho-IRS1, (**B**) phospho-Akt/Akt, and (**C**) phospho-GSK-3β protein expression. Representative images by fluorescence microscopy and FACS analysis of (**D**–**F**) intracellular and (**G**–**I**) mitochondrial ROS detection, expressed as MFI, and (**J**) acetylated-SOD2/SOD2 protein levels in EC transfected with mimic Negative Control (mimic NC), SIRT3 mimic (SIRT3^+^), mimic Negative Control and then exposed to 0.5 mM PA for 48 h (mimic NC+PA) or SIRT3 mimic before 48 h treatment with PA (SIRT3^+^+PA). Scale bars = 100 µm. Western blotting results (*n* = 5) are expressed as arbitrary units (AU) and represented as boxplots. † *p* < 0.01 vs. mimic NC; ‡ *p* < 0.001 vs. mimic NC; § *p* < 0.05 vs. mimic NC+PA; # *p* < 0.01 vs. mimic NC+PA.

Evaluation of phosphorylated-IRS1, -Akt, and -GSK-3β protein levels showed that SIRT3 overexpression was able to counteract the PA-induced IR state (*p* < 0.001 vs. mimic NC+PA) (Figure 3A–C and Figure S8).

When we assessed the content of intracellular and mitochondrial ROS during PA treatment, results showed an extensive intracellular (559 ± 18.7 vs. 406 ±.14.9 MFI, in mimic NC, *p* < 0.01) and mitochondrial (753 ± 35.1 vs. 194 ± 23.4 MFI, in mimic NC, *p* < 0.001) ROS generation which was opposed by SIRT3^+^ (SIRT3^+^+PA) (491 ± 11.9 with *p* < 0.05 and 244 ± 30.5 with *p* < 0.01 vs. mimic NC+PA, respectively) (Figure 3). Even more importantly, a reduction of acetylated-SOD2/SOD2 ratio was observed in SIRT3^+^+PA cells, supporting the deacetylation and activation of SOD2 (Figure 3 and Figure S8). ROS inducer, menadione, and the transfectant reagent were also tested as positive and negative controls, respectively (Figure S2).

### 3.4. Effects of SIRT3^+^ on PA-Induced Inflammation

**Figure 4:**
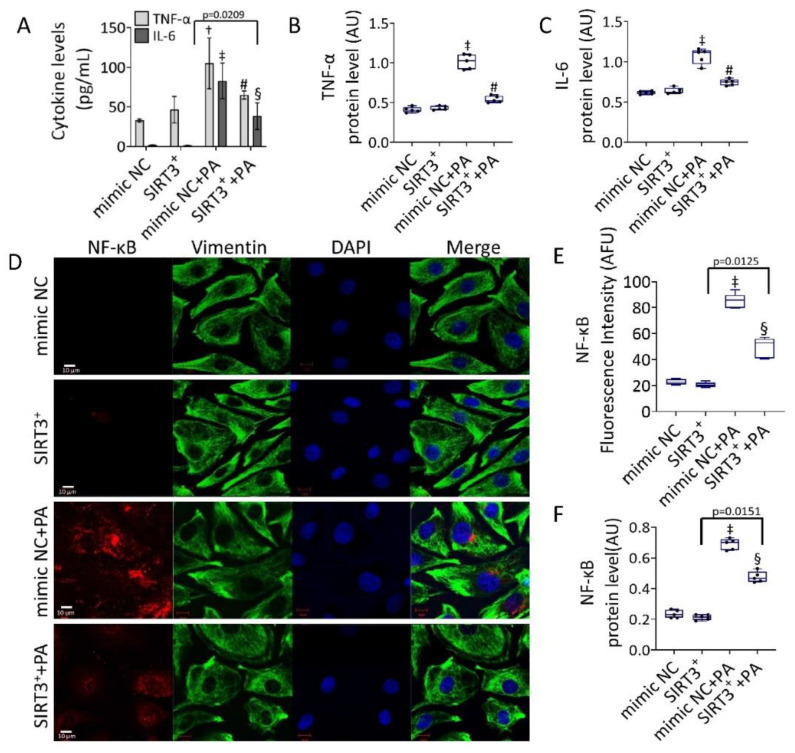
SIRT3^+^ counteracted the PA-induced inflammation. (**A**) Cytokine content and protein level of (**B**) TNF-α, and (**C**) IL-6. (**D**) Representative images of confocal laser scanning microscopy (scale bars = 10 µm). (**E**) Fluorescence intensity and (**F**) protein expression levels of NF-κB in EC transfected with mimic Negative Control (mimic NC), SIRT3 mimic (SIRT3^+^), mimic Negative Control and then exposed to 0.5 mM PA for 48 h (mimic NC+PA) or SIRT3 mimic before 48 h treatment with PA (SIRT3^+^+PA). Fluorescence analysis is reported as boxplots of arbitrary fluorescence units (AFU) of 5 independent experiments. Densitometric immunoblotting study (*n* = 5) is expressed as arbitrary units (AU). † *p* < 0.01 vs. mimic NC; ‡ *p* < 0.001 vs. mimic NC; § *p* < 0.05 vs. mimic NC+PA; # *p* < 0.01 vs. mimic NC+PA.

The efficacy of SIRT3^+^ to oppose cytokine content induced by PA was then investigated (Figure 4).

The results indicated that the protective effect of SIRT3^+^ was accompanied by its efficacy in reducing IL-6 and TNF-α levels and protein expression induced by PA (*p* < 0.01 vs. mimic NC+PA) (Figure 4A–C and Figure S9), as well as in downregulating the expression levels of NF-κB. Specifically, in SIRT3^+^+PA the upregulation of NF-κB protein was lower compared to mimic NC+PA (0.467 ± 0.04 AU vs. 0.657 ± 0.03 AU and 53.78 ± 3.14 AFU vs. 84.7 ± 5.21 AFU, *p* < 0.05) (Figure 4D–F and Figure S9).

### 3.5. SIRT3^+^ Reduced the PA-Induced Pyroptosis

**Figure 5:**
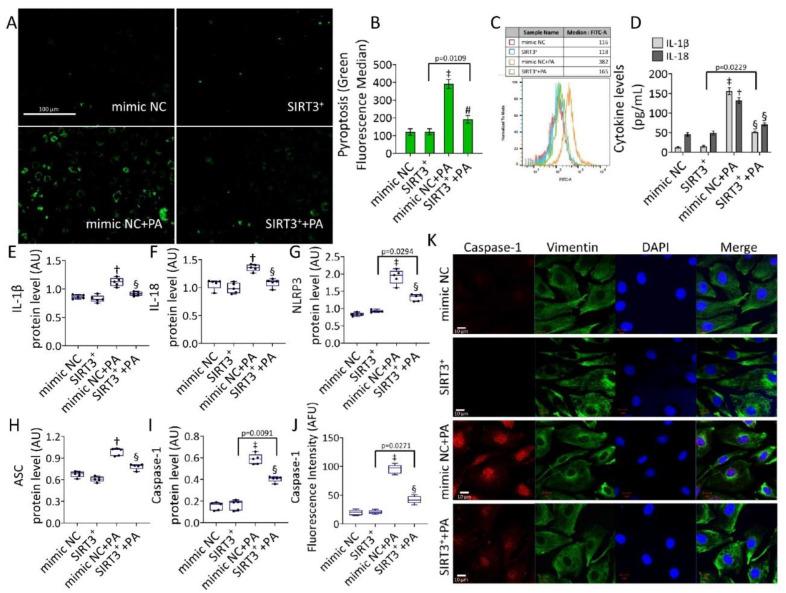
SIRT3^+^ reduced the PA-mediated pyroptosis. (**A**) Representative images and (**B**,**C**) cytometer analysis, expressed as green fluorescence median, of pyroptosis (scale bars = 100 µm). (**D**) Cytokine levels and immunoblotting analysis of (**E**) IL-1β, (**F**) IL-18, (**G**) NLRP3, (**H**) ASC, and (**I**) caspase-1. (**J**) Fluorescence intensity and (**K**) representative images of confocal laser scanning microscopy (scale bars = 10 µm) of caspase-1 in EC transfected with mimic Negative Control (mimic NC), SIRT3 mimic (SIRT3^+^), mimic Negative Control and then exposed to 0.5 mM PA for 48 h (mimic NC+PA) or SIRT3 mimic before 48 h treatment with PA (SIRT3^+^+PA). Fluorescence analysis (*n* = 5) is reported as boxplots of arbitrary fluorescence units (AFU), protein expression levels (*n* = 5) expressed as arbitrary units (AU). † *p* < 0.01 vs. mimic NC; ‡ *p* < 0.001 mimic NC; § *p* < 0.05 vs. mimic NC+PA; # *p* < 0.01 vs. mimic NC+PA.

The NF-κB signaling pathway is a crucial regulator of NLRP3 inflammasome formation. We next evaluated the occurrence of pyroptosis in PA-treated EC (Figure 5).

Results showed that pyroptosis induced by PA (*p* < 0.001) was reverted in SIRT3^+^+PA (*p* < 0.01 vs. mimic NC+PA) (Figure 5A–C). SIRT3^+^+PA attenuated both content and expression levels of IL-18 and IL-1β (*p* < 0.05 vs. mimic NC+PA) (Figure 5D–F and Figure S10), as well as NLRP3, ASC, and caspase-1 protein expression (*p* < 0.05 vs. mimic NC+PA) (Figure 5G–K and Figure S10). Pyroptosis inducer, nigericin, and the transfectant reagent were tested as positive and negative controls, respectively (Figure S3A,B). Annexin V/PI measurements revealed that the cytotoxicity induced by PA was accompanied by a weak apoptotic rate (Figure S3C,D), indicating that apoptosis is not a predominant mechanism. In detail, a reduction of live cells (72.9% ± 2.17 vs. 82.8% ± 1.98 in mimic NC, *p* < 0.05) and an increase of apoptotic cells (22.9% ± 3.96 vs. 10.0% ± 2.19 in mimic NC, *p* < 0.05) were observed. Instead, results indicated a reduction of the PA-mediated cytotoxic effects in SIRT3^+^+PA treated cells (81.0% ± 3.22 of live cells and 14.8 % ± 1.09 of apoptotic cells, *p* < 0.05 vs. mimic NC+PA) (Figure S3C,D).

### 3.6. SIRT3^+^ Reduced the Autophagy Induced by PA

**Figure 6:**
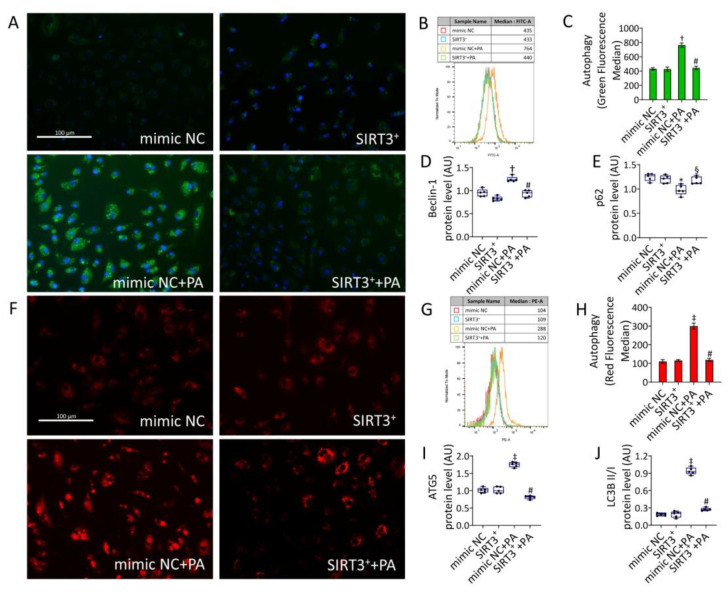
SIRT3^+^ decreased the PA-induced autophagy. Representative images of fluorescence microscopy and flow cytometry analysis of (**A**–**C**) green detection reagent and (**F**–**H**) Lysotracker Red, quantified as MFI (scale bars = 100 µm). Western blotting analysis of (**D**) beclin-1, (**E**) p62, (**I**) ATG5, and (**J**) LC3B II/I in EC transfected with mimic Negative Control (mimic NC), SIRT3 mimic (SIRT3^+^), mimic Negative Control and then exposed to 0.5 mM PA for 48 h (mimic NC+PA) or SIRT3 mimic before 48 h treatment with PA (SIRT3^+^+PA). Western blotting results (*n* = 5) are expressed as arbitrary units (AU) and represented as boxplots. † *p* < 0.01 vs. mimic NC; ‡ *p* < 0.001 vs. mimic NC; § *p* < 0.05 vs. mimic NC+PA; # *p* < 0.01 vs. mimic NC+PA.

Since autophagy is known to modulate pyroptosis [11,14], the autophagic mechanism was also investigated. As for pyroptosis, the PA-induced lysosome accumulation and autophagy (*p* < 0.001) was reduced in SIRT3^+^+PA cells (*p* < 0.01 vs. mimic NC+PA) (Figure 6).

Moreover, PA positively modulated the autophagic effector beclin-1 (*p* < 0.01), ATG5, and LC3BII (*p* < 0.001). These effects were accompanied by a decrease in p62 protein levels (*p* < 0.05). Of note, SIRT3^+^+PA led to a lower modulation of the autophagic effectors (*p* < 0.01 vs. mimic NC+PA) (Figure 6 and Figure S11). The autophagy inducer rapamycin (1 µM overnight) and the transfectant reagent were tested as positive and negative controls, respectively (Figure S4).

### 3.7. Effects of δVB on Mitochondrial ROS and Sirtuin Modulation

**Figure 7:**
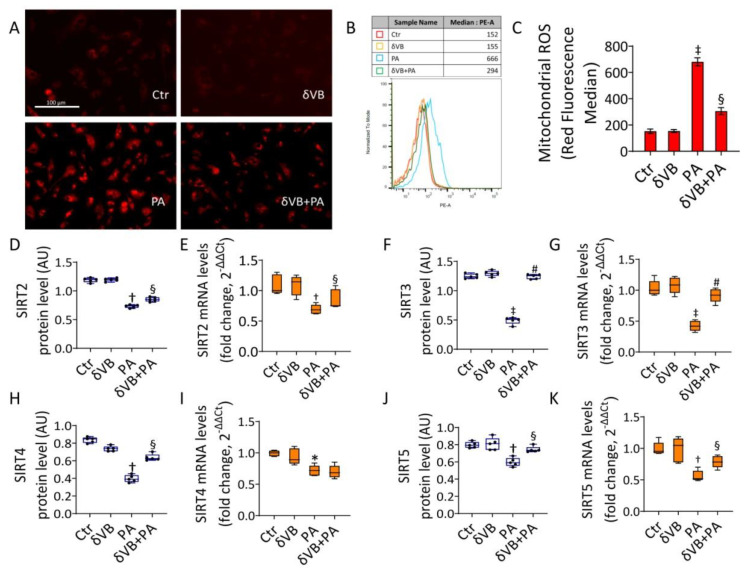
δVB reduced the PA-induced mitochondrial dysfunction. (**A**) Representative images by fluorescence microscopy (scale bars = 100 µm) and (**B**,**C**) FACS analysis of mitochondrial ROS detection (MFI). Levels of mRNA and immunoblotting analysis of (**D**,**E**) SIRT2, (**F**,**G**) SIRT3, (**H**,**I**) SIRT4, and (**J**,**K**) SIRT5 in EC exposed for 48 h to 0.5 mM δVB, 0.5 mM PA (PA), or pretreated for 16 h with δVB before 48 h PA treatment (δVB+PA). Control cells (Ctr) were treated with the corresponding highest volume of HBSS-10 mM Hepes. Western blotting results (*n* = 5) are expressed as arbitrary units (AU). mRNA levels are reported as floating bars with line representing the median ± SD (*n* = 3). * *p* < 0.05 vs. Ctr; † *p* < 0.01 vs. Ctr; ‡ *p* < 0.001 vs. Ctr; § *p* < 0.05 vs. PA; # *p* < 0.01 vs. PA.

The antioxidant δVB was tested as a possible modulator of the mitochondrial homeostasis during exposure to PA. Results showed that cell viability was not affected by treatment with δVB up to 72 h of incubation (Figure S5A). Dose-response indicated that 0.5 mM δVB was the most effective dose in reducing the cytotoxic effects of PA (*p* < 0.01 vs. PA), acting on the IRS1/Akt pathway (Figures S5B,D and S14). Pretreatment with 0.5 mM δVB before exposure to PA (δVB+PA) attenuated the mitochondrial ROS cascade (294 ± 17.7 vs. 666 ± 31.4 MFI in PA, *p* < 0.05) (Figure 7A–C) and upregulated SIRT2, SIRT3, SIRT4, and SIRT5 protein and mRNA, with the highest effect on SIRT3 (*p* < 0.01) (Figure 7F,G and Figure S12).

### 3.8. δVB as a Modulator of PA-Induced Pyroptosis and Autophagy

**Figure 8:**
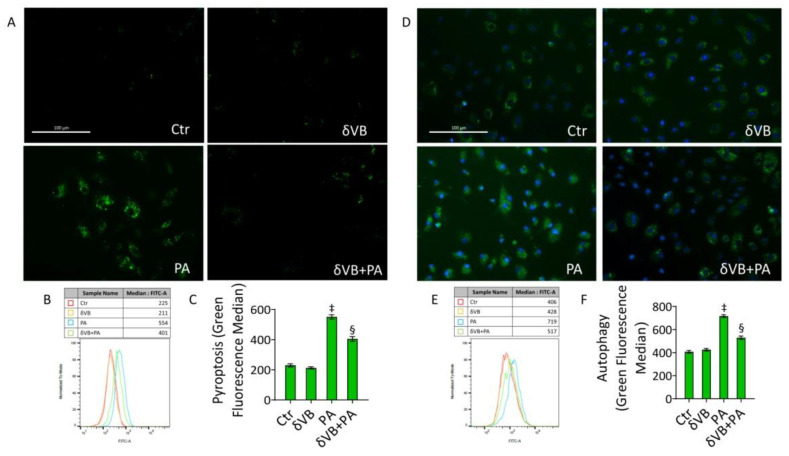
δVB effects on PA-induced pyroptosis and autophagy. Representative images by fluorescence microscopy and flow cytometry analysis of (**A**–**C**) pyroptosis and (**D**–**F**) autophagy, expressed as green median fluorescence ± SD (*n* = 3), in EC exposed for 48 h to 0.5 mM δVB, 0.5 mM PA (PA), or pretreated for 16 h with δVB before 48 h PA treatment (δVB + PA). Control cells (Ctr) were treated with the corresponding highest volume of HBSS-10 mM Hepes. Scale bars = 100 µm. ‡ *p* < 0.001 vs. Ctr; § *p* < 0.05 vs. PA.

Results indicated that δVB was also effective in reducing pyroptosis (401 ± 33.8 vs. 554 ± 36.1 MFI in PA, *p* < 0.05) and autophagy (517 ± 34.5 vs. 719 ± 27.6 MFI in PA, *p* < 0.05) (Figure 8), suggesting the efficacy of this antioxidant as a natural modulator of PA-induced pyroptosis and autophagy.

## 4. Discussion

Here, we investigated the role of mitochondrial sirtuins in the protection of human endothelial cells against PA-induced IR. The PA-induced mitochondrial dysfunction led to pyroptosis, autophagy, and apoptosis. Transfection with SIRT3 mimics reverted PA-induced ROS accumulation, inflammatory-related pyroptosis, and autophagy. Furthermore, the modulation of SIRT3 levels and the improvement of PA-induced mitochondrial dysfunction have been achieved using the antioxidant δVB.

Among histone deacetylases, mitochondrial SIRT2, SIRT3, SIRT4, and SIRT5 are key metabolic enzymes controlling mitochondrial homeostasis and response to oxidative stress [3,27,28,29]. Compelling evidence indicates that mitochondrial sirtuins are involved in the regulation of endothelial function given their ability to regulate energy metabolism, ROS production, and cellular redox state [2]. SIRT3, the main mitochondrial deacetylase, regulates multiple metabolic pathways and its expression is reduced in diabetes [2]. Moreover, SIRT3 activation improved IR while its inhibition precipitated IR in adipocytes [30]. Recent findings suggest that endothelial IR induced by SIRT3 deletion could reprogram endothelial cell metabolism, shifting metabolism from oxygen-independent glycolysis to oxygen-dependent oxidative phosphorylation [31,32]. Downregulation of SIRT3 by siRNA reduced insulin response in human umbilical vein endothelial cells (HUVECs), whereas overexpression improved IR induced by palmitate, thus suggesting an association between SIRT3 and insulin sensitivity [32]. Here, overexpression of SIRT3 in endothelial TeloHAEC cells abolished mitochondrial ROS generation caused by PA and counteracted IR injuries through the attenuation of inflammation, which then flowed to the inflammatory form of programmed cell death, pyroptosis. Interestingly, PA induced only a weak apoptotic cell death rate, suggesting that pyroptosis represents the principal cellular phenomenon during PA-induced IR. Calorie restriction and exercise-mediated weight loss decreased mRNA levels of NLRP3 and IL-1β, and improved insulin sensitivity in diabetic subjects [33], demonstrating that pyroptosis contributes to the pathogenesis of IR and diabetes by mediating inflammation and β-cell destruction. The regulation of SIRT2, SIRT3, and SIRT4 expression is known to relate to inflammation and pyroptosis cell death [34,35,36]. To date, the concept of endothelial IR has not been clinically established because only a small number of clinical studies have shown the actual response to insulin in endothelial cells of patients with metabolic disorders [37,38,39,40,41,42].

The use of naturally occurring compounds in lifestyle and nutrition improves and/or prevents aging-related diseases such as IR, diabetes, and its vascular complications [19,43]. Recent advances in small metabolites have opened novel avenues in the metabolic route of betaines, metabolites with a quaternary ammonium group involved in the regulation of metabolic homeostasis such as protection against osmotic stress. One such compound, δVB, has been associated with positive health effects (fetal brain development, insulin secretion, and reduced cancer risk) and some negative health outcomes associated with a high-fat diet (cardiovascular disease and fatty liver disease) [44], suggesting that investigations into the metabolic route and role of this novel betaine are still necessary. δVB, at least partially synthesized by gut microbes, has been reported to be present in certain food sources, including milk and meat, and the kinds of marine algae used as foodstuffs [25,44].

In vitro evidence suggests that δVB prevents endothelial damage related to diabetes [19] and displays antineoplastic effects in colon cancer cells and head and neck squamous cell carcinomas [20,21,22]. In the human and mouse heart, δVB affected energy metabolism, showing a similar effect to that of meldonium, a cardioprotective drug used to improve cardiac function after ischemia [16,17]. Clinical studies revealed that treatment of type 2 diabetes with metformin increased the δVB concentration in human serum [45]. Furthermore, increased plasma levels of δVB and other betainized compounds as a consequence of a diet rich in whole grains were correlated with improved insulin resistance and insulin secretion [16,18]. Additionally, δVB was demonstrated to improve glucose tolerance and insulin tolerance in mice [7], suggesting that the higher δVB concentration in plasma might exert some protection against the diabetogenic Western diet. In line with previous data [19], δVB did not show cytotoxicity up to 1 mM concentration and protected EC against PA-induced IR by limiting the inhibitory phosphorylation of IRS1 and restoring phosphorylated Akt expression levels. In particular, δVB reduced oxidative stress and cytokine content, restored the expression levels of SIRT3, and attenuated pyroptosis and autophagy. The beneficial effects mediated by δVB could be related, at least in part, to its ability to positively modulate SIRT3 expression levels, indicating δVB as a possible antioxidant in the prevention of IR. However, it cannot be ruled out that other molecular targets may be involved in this molecular mechanism. Further studies are undoubtedly necessary for a deeper understanding of the molecular pathway through which SIRT3 controls endothelial redox state during IR, in order to develop novel antioxidant-targeted preventive approaches.

## 5. Conclusions

Mitochondrial homeostasis is a crucial feature to contrast endothelial injuries related to IR. SIRT3 overexpression improved the endothelial oxidative status and reduced PA-induced programmed cell death, pyroptosis, apoptosis, and autophagy. Furthermore, the antioxidant betaine, δVB, acted as a promising SIRT3 modulator in defining future and innovative preventive strategies for IR.

## Data Availability

The data presented in this study are available from the corresponding author upon request.

## Supplementary Materials

The following supporting information can be downloaded at: https://www.mdpi.com/article/10.3390/antiox11081611/s1, Figure S1: PA-induction of insulin resistance; Figure S2: Oxidative stress controls; Figure S3: Pyroptosis controls and cell death evaluation; Figure S4: Autophagy controls; Figure S5: δVB effects on PA-related insulin resistance; Figure S6: Image reporting untouched, uncropped gels related to Figure 1; Figure S7: Image reporting untouched, uncropped gels related to Figure 2; Figure S8: Image reporting untouched, uncropped gels related to Figure 3; Figure S9: Image reporting untouched, uncropped gels related to Figure 4; Figure S10: Image reporting untouched, uncropped gels related to Figure 5; Figure S11: Image reporting untouched, uncropped gels related to Figure 6; Figure S12: Image reporting untouched, uncropped gels related to Figure 7; Figure S13: Image reporting untouched, uncropped gels related to Figure S1; Figure S14: Image reporting untouched, uncropped gels related to Figure S5.

## References

[1] He X., Zeng H., Chen J.X. (2019). Emerging role of SIRT3 in endothelial metabolism, angiogenesis, and cardiovascular disease. J. Cell. Physiol..

[2] Kane A.E., Sinclair D.A. (2018). Sirtuins and NAD^+^ in the development and treatment of metabolic and cardiovascular diseases. Circ. Res..

[3] Colloca A., Balestrieri A., Anastasio C., Balestrieri M.L., D’Onofrio N. (2022). Mitochondrial sirtuins in chronic degenerative diseases: New metabolic targets in colorectal cancer. Int. J. Mol. Sci..

[4] D’Onofrio N., Vitiello M., Casale R., Servillo L., Giovane A., Balestrieri M.L. (2015). Sirtuins in vascular diseases: Emerging roles and therapeutic potential. Biochim. Biophys. Acta.

[5] D’Onofrio N., Servillo L., Balestrieri M.L. (2018). SIRT1 and SIRT6 Signaling pathways in cardiovascular disease protection. Antioxid. Redox Signal.

[6] Riehle C., Abel E.D. (2016). Insulin signaling and heart failure. Circ. Res..

[7] De Meyts P., Feingold K.R., Anawalt B., Boyce A., Chrousos G., de Herder W.W., Dhatariya K., Dungan K., Hershman J.M., Hofland J., Kalra S. (2016). The Insulin Receptor and Its Signal Transduction Network.

[8] Zhang Q.J., McMillin S.L., Tanner J.M., Palionyte M., Abel E.D., Symons J.D. (2009). Endothelial nitric oxide synthase phosphorylation in treadmill-running mice: Role of vascular signalling kinases. J. Physiol..

[9] Gélinas D.S., Bernatchez P.N., Rollin S., Bazan N.G., Sirois M.G. (2002). Immediate and delayed VEGF-mediated NO synthesis in endothelial cells: Role of PI3K, PKC and PLC pathways. Br. J. Pharmacol..

[10] Masaki N., Ido Y., Yamada T., Yamashita Y., Toya T., Takase B., Hamburg N.M., Adachi T. (2019). Endothelial insulin resistance of freshly isolated arterial endothelial cells from radial sheaths in patients with suspected coronary artery disease. J. Am. Heart Assoc..

[11] Sharma D., Kanneganti T.D. (2016). The cell biology of inflammasomes: Mechanisms of inflammasome activation and regulation. J. Cell Biol..

[12] Zheng D., Shi Z., Yang M., Liang B., Zhou X., Jing L., Sun Z. (2021). NLRP3 inflammasome-mediated endothelial cells pyroptosis is involved in decabromodiphenyl ethane-induced vascular endothelial injury. Chemosphere.

[13] Liu H., Tang D., Zhou X., Yang X., Chen A.F. (2020). PhospholipaseCγ1/calcium dependent membranous localization of Gsdmd-N drives endothelial pyroptosis, contributing to lipopolysaccharide-induced fatal outcome. Am. J. Physiol. Heart Circ. Physiol..

[14] Li H.B., Jin C., Chen Y., Flavell R.A. (2014). Inflammasome activation and metabolic disease progression. Cytokine Growth Factor Rev..

[15] Jo E.K., Kim J.K., Shin D.M., Sasakawa C. (2016). Molecular mechanisms regulating NLRP3 inflammasome activation. Cell. Mol. Immunol..

[16] Kärkkäinen O., Lankinen M.A., Vitale M., Jokkala J., Leppänen J., Koistinen V., Lehtonen M., Giacco R., Rosa-Sibakov N., Micard V. (2018). Diets rich in whole grains increase levels of betainized compounds associated with glucose metabolism. Am. J. Clin. Nutr..

[17] Kärkkäinen O., Tuomainen T., Koistinen V., Tuomainen M., Leppänen J., Laitinen T., Lehtonen M., Rysä J., Auriola S., Poso A. (2018). Whole grain intake associated molecule 5- aminovaleric acid betaine decreases β-oxidation of fatty acids in mouse cardiomyocytes. Sci. Rep..

[18] Tuomainen M., Kärkkäinen O., Leppänen J., Auriola S., Lehtonen M., Savolainen M.J., Hermansen K., Risérus U., Åkesson B., Thorsdottir I. (2019). Quantitative assessment of betainized compounds and associations with dietary and metabolic biomarkers in the randomized study of the healthy Nordic diet (SYSDIET). Am. J. Clin. Nutr..

[19] D’Onofrio N., Balestrieri A., Neglia G., Monaco A., Tatullo M., Casale R., Limone A., Balestrieri M.L., Campanile G. (2019). Antioxidant and anti-inflammatory activities of buffalo milk δ-Valerobetaine. J. Agric. Food Chem..

[20] D’Onofrio N., Cacciola N.A., Martino E., Borrelli F., Fiorino F., Lombardi A., Neglia G., Balestrieri M.L., Campanile G. (2020). ROS-mediated apoptotic cell death of human colon cancer LoVo cells by milk δ-valerobetaine. Sci. Rep..

[21] D’Onofrio N., Martino E., Mele L., Colloca A., Maione M., Cautela D., Castaldo D., Balestrieri M.L. (2021). Colorectal cancer apoptosis induced by dietary δ-valerobetaine involves PINK1/Parkin dependent-mitophagy and SIRT3. Int. J. Mol. Sci..

[22] D’Onofrio N., Mele L., Martino E., Salzano A., Restucci B., Cautela D., Tatullo M., Balestrieri M.L., Campanile G. (2020). Synergistic effect of dietary betaines on SIRT1-mediated apoptosis in human oral squamous cell carcinoma Cal 27. Cancers.

[23] Tatullo M., Marrelli B., Benincasa C., Aiello E., Amantea M., Gentile S., Leonardi N., Balestrieri M.L., Campanile G. (2022). Potential impact of functional biomolecules-enriched foods on human health: A randomized controlled clinical trial. Int. J. Med. Sci..

[24] Servillo L., Giovane A., Cautela D., Castaldo D., Balestrieri M.L. (2014). Where does N(ε)-trimethyllysine for the carnitine biosynthesis in mammals come from?. PLoS ONE.

[25] Servillo L., D’Onofrio N., Giovane A., Casale R., Cautela D., Castaldo D., Iannaccone F., Neglia G., Campanile G., Balestrieri M.L. (2018). Ruminant meat and milk contain δ-valerobetaine, another precursor of trimethylamine N-oxide (TMAO) like γ-butyrobetaine. Food Chem..

[26] Wang Y., Fu W., Xue Y., Lu Z., Li Y., Yu P., Yu X., Xu H., Sui D. (2021). Ginsenoside Rc ameliorates endothelial insulin resistance via upregulation of angiotensin-converting enzyme 2. Front. Pharmacol..

[27] Teixeira C.S.S., Cerqueira N.M.F.S.A., Gomes P., Sousa S.F. (2020). A molecular perspective on sirtuin activity. Int. J. Mol. Sci..

[28] Shahgaldi S., Kahmini F.R. (2021). A comprehensive review of Sirtuins: With a major focus on redox homeostasis and metabolism. Life Sci..

[29] Liu G., Park S.H., Imbesi M., Nathan W.J., Zou X., Zhu Y., Jiang H., Parisiadou L., Gius D. (2017). Loss of NAD-dependent protein deacetylase sirtuin-2 alters mitochondrial protein acetylation and dysregulates mitophagy. Antioxid. Redox Signal.

[30] Lee A.Y., Christensen S.M., Duong N., Tran Q.A., Xiong H.M., Huang J., James S., Vallabh D., Talbott G., Rose M. (2022). Sirt3 pharmacologically promotes insulin sensitivity through PI3/AKT/mTOR and their downstream pathway in adipocytes. Int. J. Mol. Sci..

[31] Yang L., Zhang J., Xing W., Zhang X., Xu J., Zhang H., Chen L., Ning X., Ji G., Li J. (2016). SIRT3 deficiency induces endothelial insulin resistance and blunts endothelial-dependent vasorelaxation in mice and human with obesity. Sci. Rep..

[32] He X., Zeng H., Chen S.T., Roman R.J., Aschner J.L., Didion S., Chen J.X. (2017). Endothelial specific SIRT3 deletion impairs glycolysis and angiogenesis and causes diastolic dysfunction. J. Mol. Cell Cardiol..

[33] Vandanmagsar B., Youm Y.H., Ravussin A., Galgani J.E., Stadler K., Mynatt R.L., Ravussin E., Stephens J.M., Dixit V.D. (2011). The NLRP3 inflammasome instigates obesity-induced inflammation and insulin resistance. Nat. Med..

[34] Luo H., Mu W.C., Karki R., Chiang H.H., Mohrin M., Shin J.J., Ohkubo R., Ito K., Kanneganti T.D., Chen D. (2019). Mitochondrial stress-initiated aberrant activation of the NLRP3 inflammasome regulates the functional deterioration of hematopoietic stem cell aging. Cell Rep..

[35] Xu X., Zhang L., Hua F., Zhang C., Zhang C., Mi X., Qin N., Wang J., Zhu A., Qin Z. (2021). FOXM1-activated SIRT4 inhibits NF-κB signaling and NLRP3 inflammasome to alleviate kidney injury and podocyte pyroptosis in diabetic nephropathy. Exp. Cell Res..

[36] Guan C., Huang X., Yue J., Xiang H., Shaheen S., Jiang Z., Tao Y., Tu J., Liu Z., Yao Y. (2021). SIRT3-mediated deacetylation of NLRC4 promotes inflammasome activation. Theranostics.

[37] Tabit C.E., Shenouda S.M., Holbrook M., Fetterman J.L., Kiani S., Frame A.A., Kluge M.A., Held A., Dohadwala M.M., Gokce N. (2013). Protein kinase C-β contributes to impaired endothelial insulin signaling in humans with diabetes mellitus. Circulation.

[38] Bretòn-Romero R., Feng B., Holbrook M., Farb M.G., Fetterman J.L., Linder E.A., Berk B.D., Masaki N., Weisbrod R.M., Inagaki E. (2016). Endothelial dysfunction in human diabetes is mediated by Wnt5a-JNK signaling. Arterioscler. Thromb. Vasc. Biol..

[39] Straub L.G., Efthymiou V., Grandl G., Balaz M., Challa T.D., Truscello L., Horvath C., Moser C., Rachamin Y., Arnold M. (2019). Antioxidants protect against diabetes by improving glucose homeostasis in mouse models of inducible insulin resistance and obesity. Diabetologia.

[40] Gong L., Guo S., Zou Z. (2020). Resveratrol ameliorates metabolic disorders and insulin resistance in high-fat diet-fed mice. Life Sci..

[41] Alam M.A., Subhan N., Rahman M.M., Uddin S.J., Reza H.M., Sarker S.D. (2014). Effect of citrus flavonoids, naringin and naringenin, on metabolic syndrome and their mechanisms of action. Adv. Nutr..

[42] MacDonald-Ramos K., Michán L., Martínez-Ibarra A., Cerbón M. (2021). Silymarin is an ally against insulin resistance: A review. Ann. Hepatol..

[43] Gurau F., Baldoni S., Prattichizzo F., Espinosa E., Amenta F., Procopio A.D., Albertini M.C., Bonafè M., Olivieri F. (2018). Antisenescence compounds: A potential nutraceutical approach to healthy aging. Ageing Res. Rev..

[44] Haikonen R., Kärkkäinen O., Koistinen V., Hanhineva K. (2022). Diet- and microbiota-related metabolite, 5-aminovaleric acid betaine (5-AVAB), in health and disease. Trends Endocrinol. Metab..

[45] Adam J., Brandmaier S., Leonhardt J., Scheerer M.F., Mohney R.P., Xu T., Bi J., Rotter M., Troll M., Chi S. (2016). Metformin effect on nontargeted metabolite profiles in patients with type 2 diabetes and in multiple murine tissues. Diabetes.

