# Supplementary material for: SIRT3 Modulates Endothelial Mitochondrial Redox State during Insulin Resistance
Source: Antioxidants (Basel). 2022 Aug 19;11(8):1611. doi: 10.3390/antiox11081611 (PMC9404744; doi:10.3390/antiox11081611)
Supplement: Supplementary file 1 [file antioxidants-11-01611-s001.zip › antioxidants-1846432-supplementary.pdf]

SIRT3 modulates endothelial mitochondrial redox state during insulin resistance

Elisa Martino[1], Anna Balestrieri[2], Camilla Anastasio[1], Martina Maione[1], Luigi Mele[3], Domenico Cautela[4], Giuseppe Campanile[5], Maria Luisa Balestrieri[1,*], Nunzia D'Onofrio[1]

[1]Department of Precision Medicine, University of Campania Luigi Vanvitelli, Via L. De Crecchio 7, 80138 Naples, Italy

[2]Food Safety Department, Istituto Zooprofilattico Sperimentale del Mezzogiorno, Via Salute, 2, 80055 Portici, Italy

[3]Department of Experimental Medicine, University of Campania Luigi Vanvitelli, Via Luciano Armanni 5, 80138 Naples, Italy

[4]Stazione Sperimentale per le Industrie delle Essenze e dei Derivati dagli Agrumi (SSEA) – Azienda Speciale CCIAA di Reggio Calabria, 89125, Reggio Calabria, Italy

[5]Department of Veterinary Medicine and Animal Production, University of Naples Federico II, Via F. Delpino 1, 80137 Naples, Italy

*Correspondence: marialuisa.balestrieri@unicampania.it; Tel.: +39-081-5665865

SUPPLEMENTARY MATERIALS

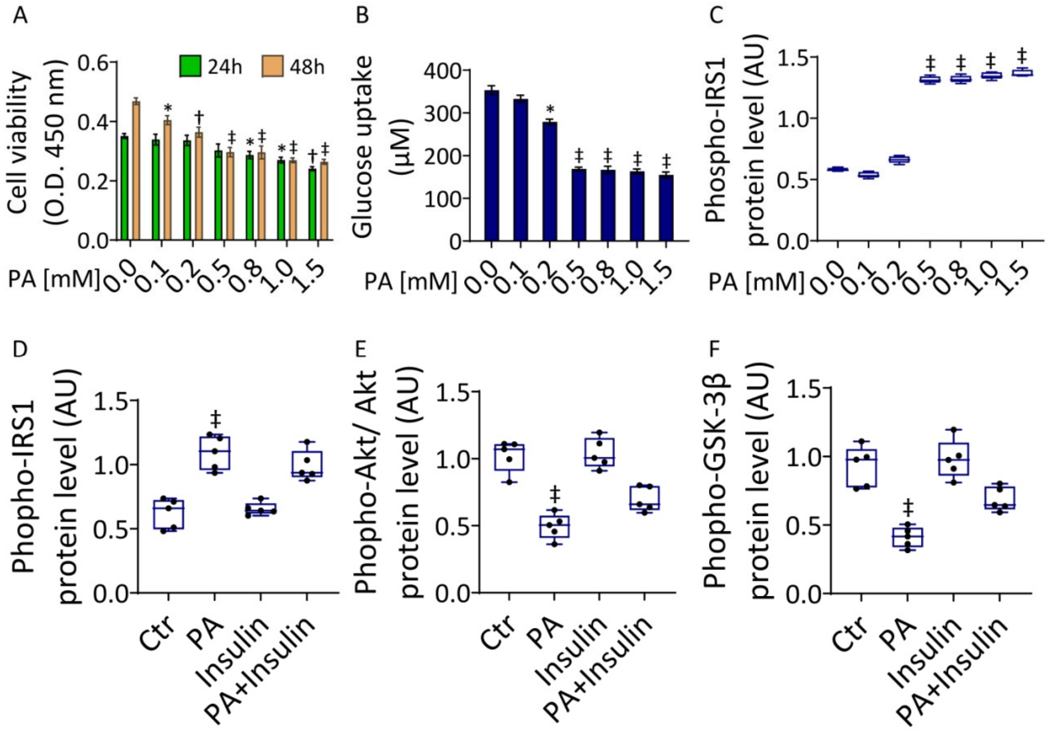

Figure S1: *PA-induction of insulin resistance.* (A) EC viability after exposure to different concentrations of palmitic acid, PA (0-1.5 mM), for 24 and 48 h. Cell viability was reported as optical density (O.D.) values. The effects of PA on (B) glucose uptake and (C) phospho-IRS1 protein expression levels were detected on EC treated with PA (0-1.5 mM) for 48 h. Immunoblotting analysis of (D) phospho-IRS1, (E) phospho-Akt/Akt and (F) phospho-GSK-3β in EC exposed to 48 h 0.5 mM PA, 100 nM insulin or to PA followed by 30 min incubation with insulin (PA+Insulin). Control cells (Ctr) were treated with the corresponding highest volume of HBSS-10 mM Hepes. Western blotting results (n = 5) are expressed as arbitrary units (AU) and represented as boxplots. * $p < 0.05$ *vs* 0 mM; † $p < 0.01$ *vs* 0 mM; ‡ $p < 0.001$ *vs* 0 mM or Ctr.

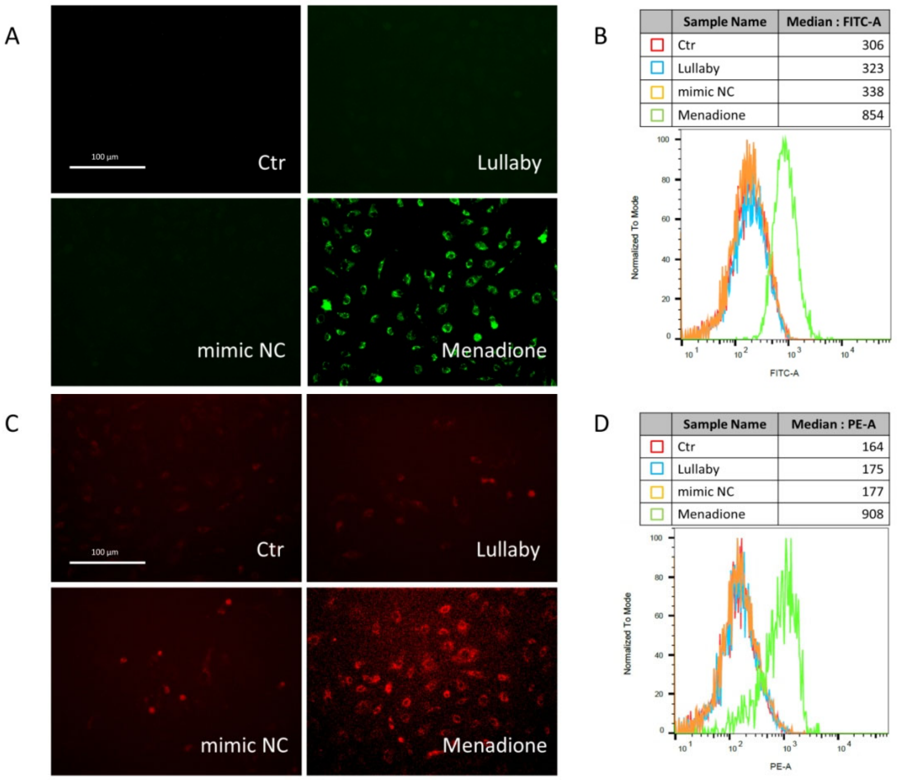

Figure S2: *Oxidative stress controls.* Representative images and cytometer analyses, expressed as green or red fluorescence median, of ROS detection in EC treated for 1 h with 50 µM menadione, the ROS inducer, with the empty transfection reagent (Lullaby) or transfected with mimic Negative Control (mimic NC) and stained with (A,B) CellROX or with (C,D) MitoSOX Red probes. Control cells (Ctr) were treated with the corresponding highest volume of HBSS-10 mM Hepes. Scale bars = 100 µm.

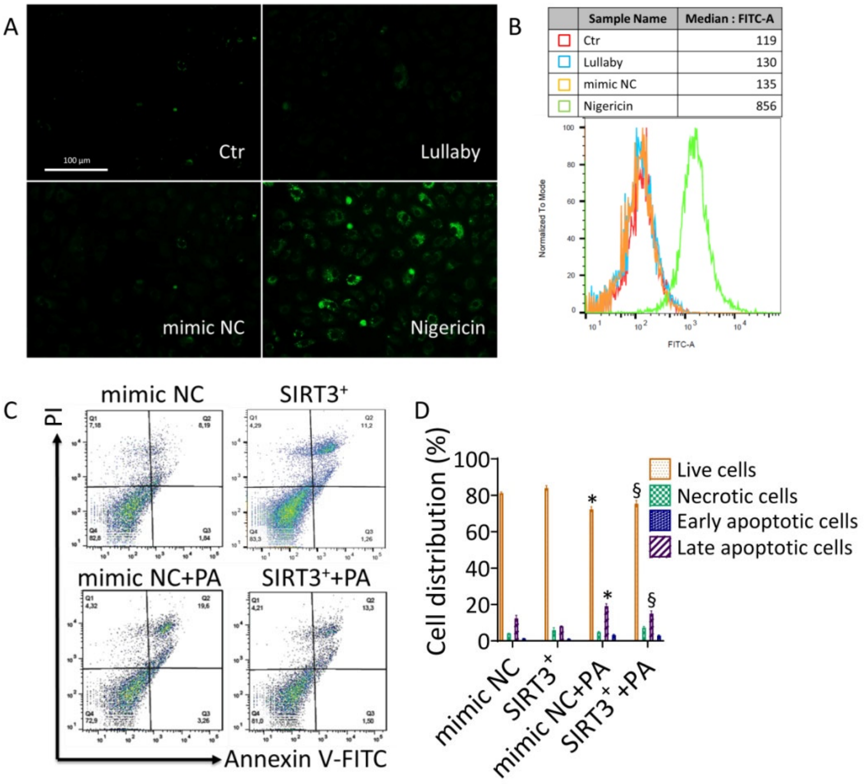

Figure S3: *Pyroptosis controls and cell death evaluation.* (A) Representative images and (B) cytometer analysis, expressed as green fluorescence median, of pyroptosis detection in EC treated for 16 h with 5 µM nigericin, the pyroptosis inducer, with the empty transfection reagent (Lullaby) or transfected with mimic Negative Control (mimic NC). Control cells (Ctr) were treated with the corresponding highest volume of HBSS-10 mM Hepes. Scale bars = 100 µm. (C) Representative dot plots and (D) analysis of Annexin V-FITC and Propidium Iodide (PI)-stained EC transfected with mimic Negative Control (mimic NC), SIRT3 mimic ($SIRT3^{+}$), mimic Negative Control before exposure to PA (mimic NC+PA) or SIRT3 mimic before 48 h treatment with PA ($SIRT3^{+}$+PA). Lower left, viable cells; upper left, necrotic cells; lower right, early apoptotic cells; upper right, late apoptotic cells. * $p < 0.05$ *vs* mimic NC; § $p < 0.05$ *vs* mimic NC+PA.

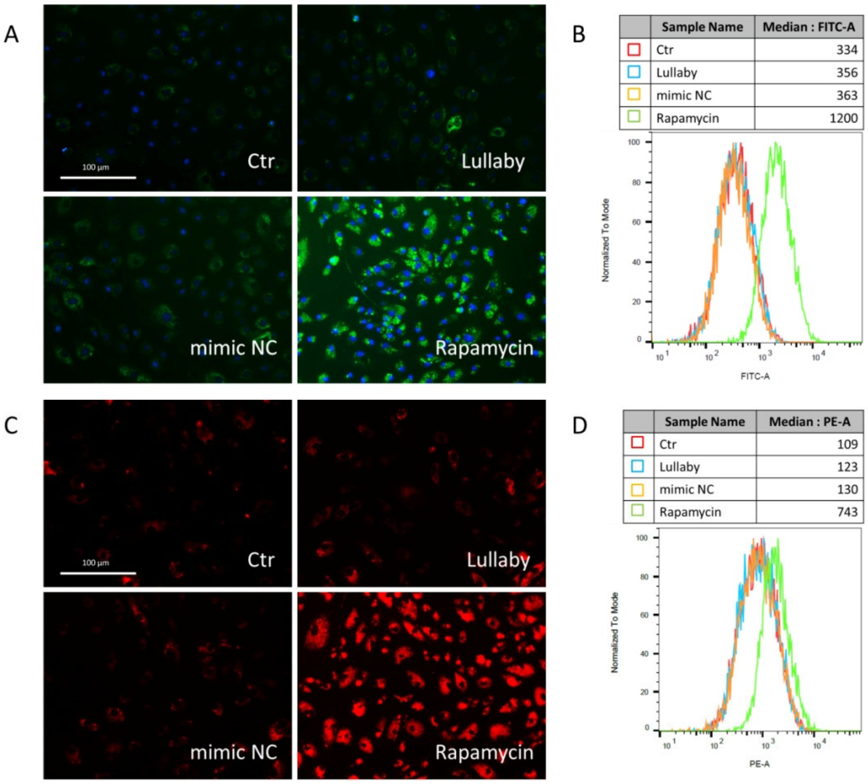

Figure S4: *Autophagy controls.* Representative images and cytometer analyses, expressed as green or red fluorescence median, of autophagy in EC treated for 16 h with 1 µM rapamycin, autophagy inducer, with the empty transfection reagent (Lullaby) or transfected with mimic Negative Control (mimic NC) and stained with (A,B) Green detection reagent or with (C,D) Lysotracker Red probe. Control cells (Ctr) were treated with the corresponding highest volume of HBSS-10 mM Hepes. Scale bars = 100 µm.

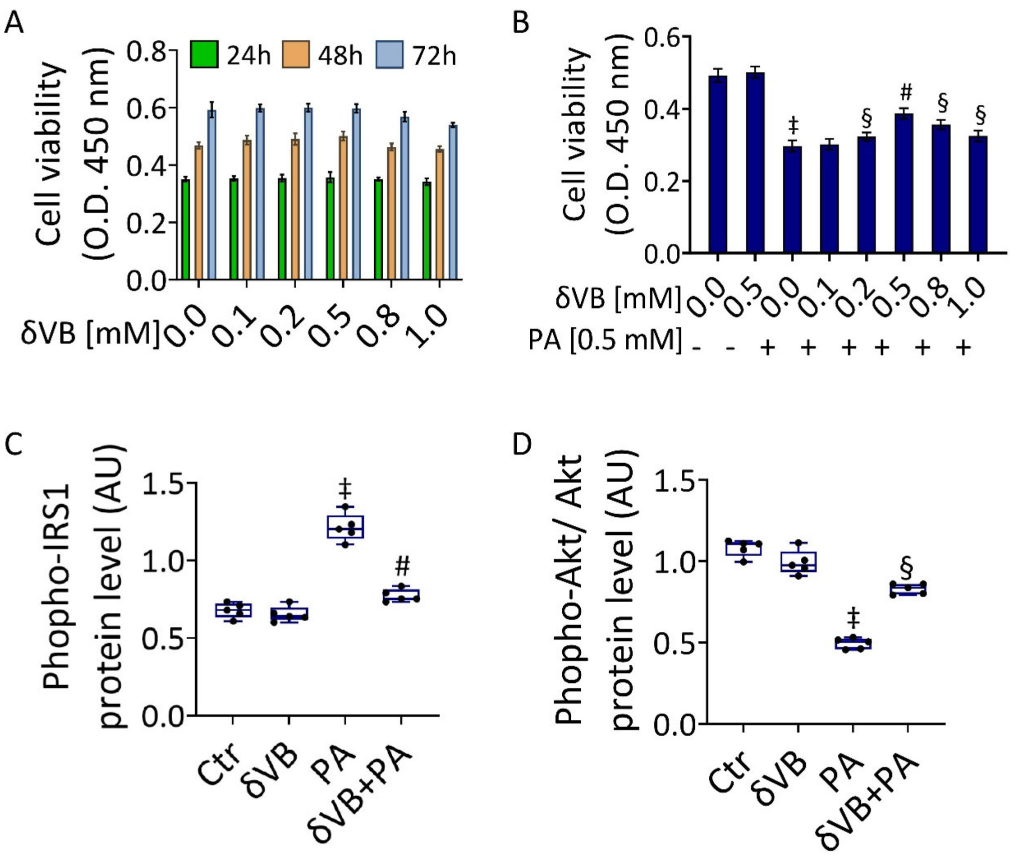

Figure S5: *δVB effects on PA-related insulin resistance.* EC viability after exposure to different concentrations of (A) δVB (0-1.0 mM) up to 72 h and (B) δVB (0-1.0 mM) for 16 h before 48 h incubation with 0.5 mM PA. Cell viability reported as optical density (O.D.) values. EC were exposed to 0.5 mM δVB, 0.5 mM PA (PA) or combined δVB+PA for 48 h and (C) phospho-IRS1 and (D) phospho-Akt/Akt protein levels assessed by immunoblotting analysis. Control cells (0 mM or Ctr) were treated with the corresponding highest volume of HBSS-10 mM Hepes. Western blotting results ($n = 5$) are expressed as arbitrary units (AU) and represented as boxplots. ‡ $p < 0.001$ *vs* 0 mM or Ctr; § $p < 0.05$ *vs* PA; # $p < 0.01$ *vs* PA.

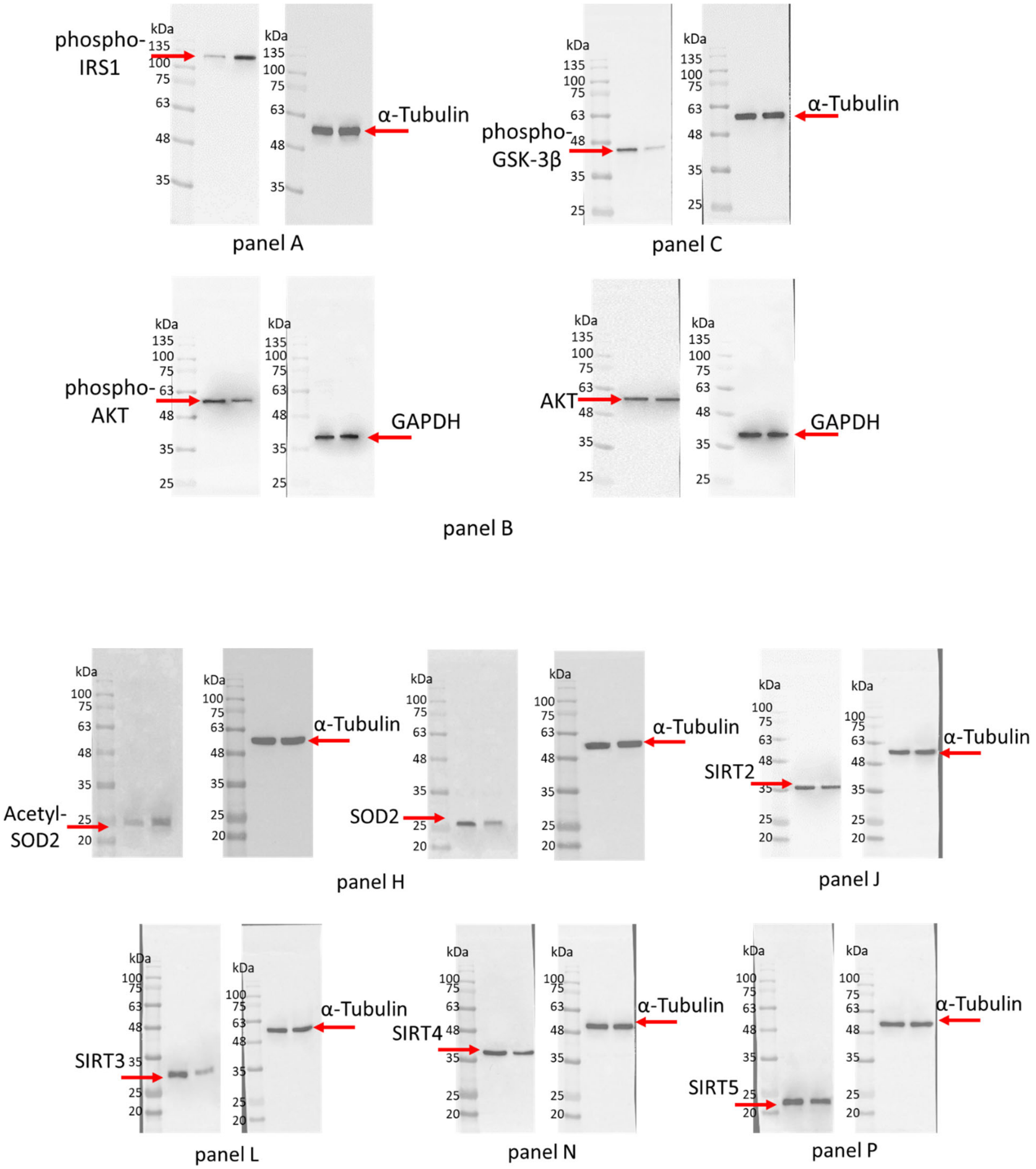

Figure S6: Image reporting untouched, uncropped gels related to Figure 1.

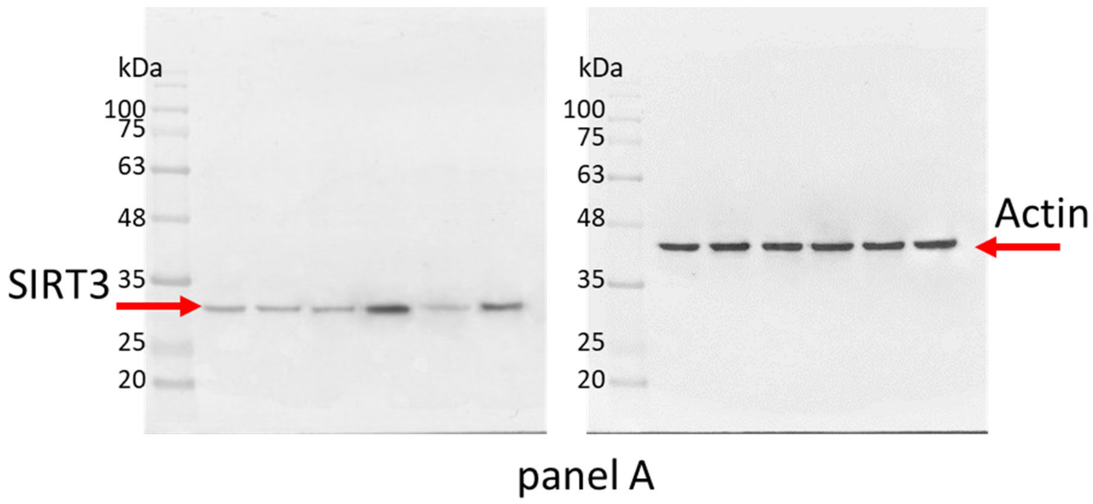

Figure S7: Image reporting untouched, uncropped gels related to Figure 2.

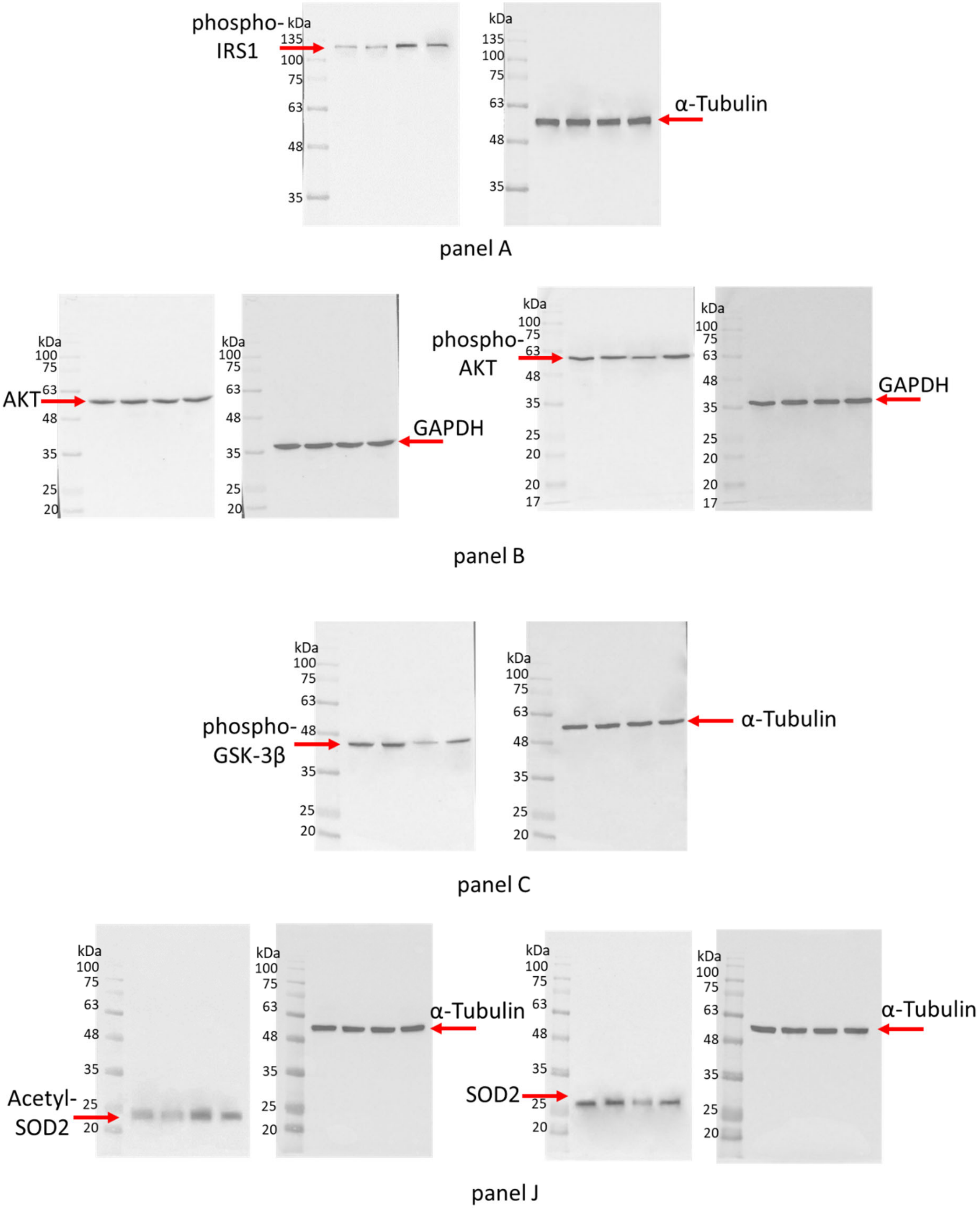

Figure S8: Image reporting untouched, uncropped gels related to Figure 3.

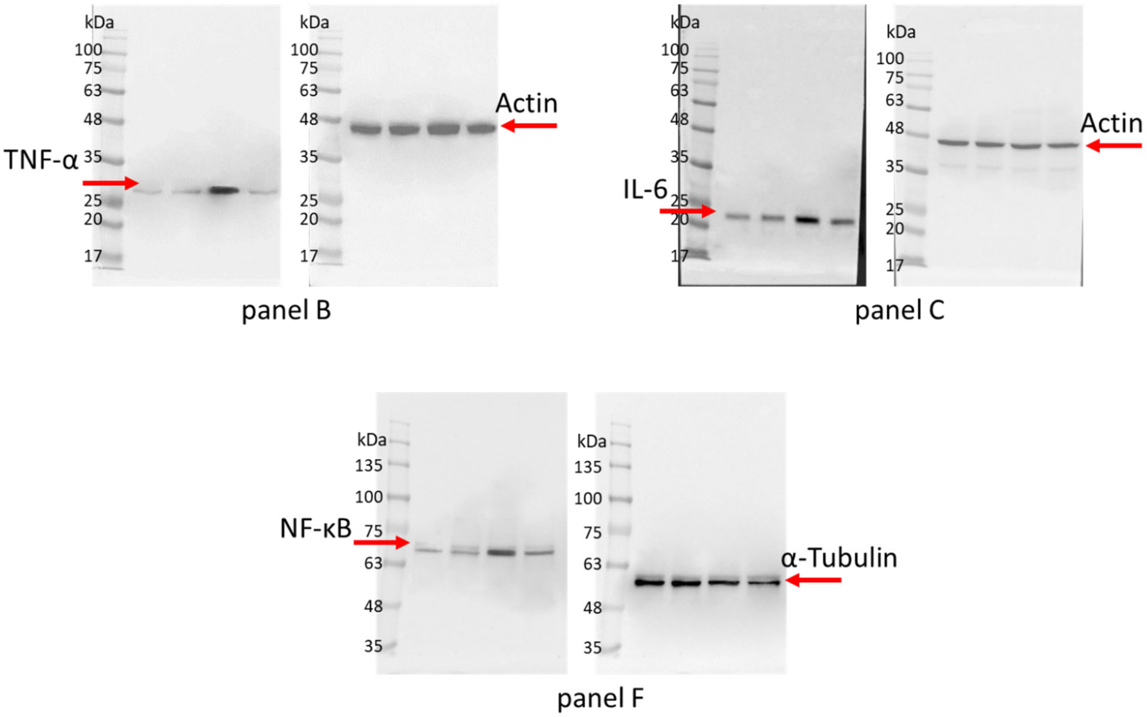

Figure S9: Image reporting untouched, uncropped gels related to Figure 4.

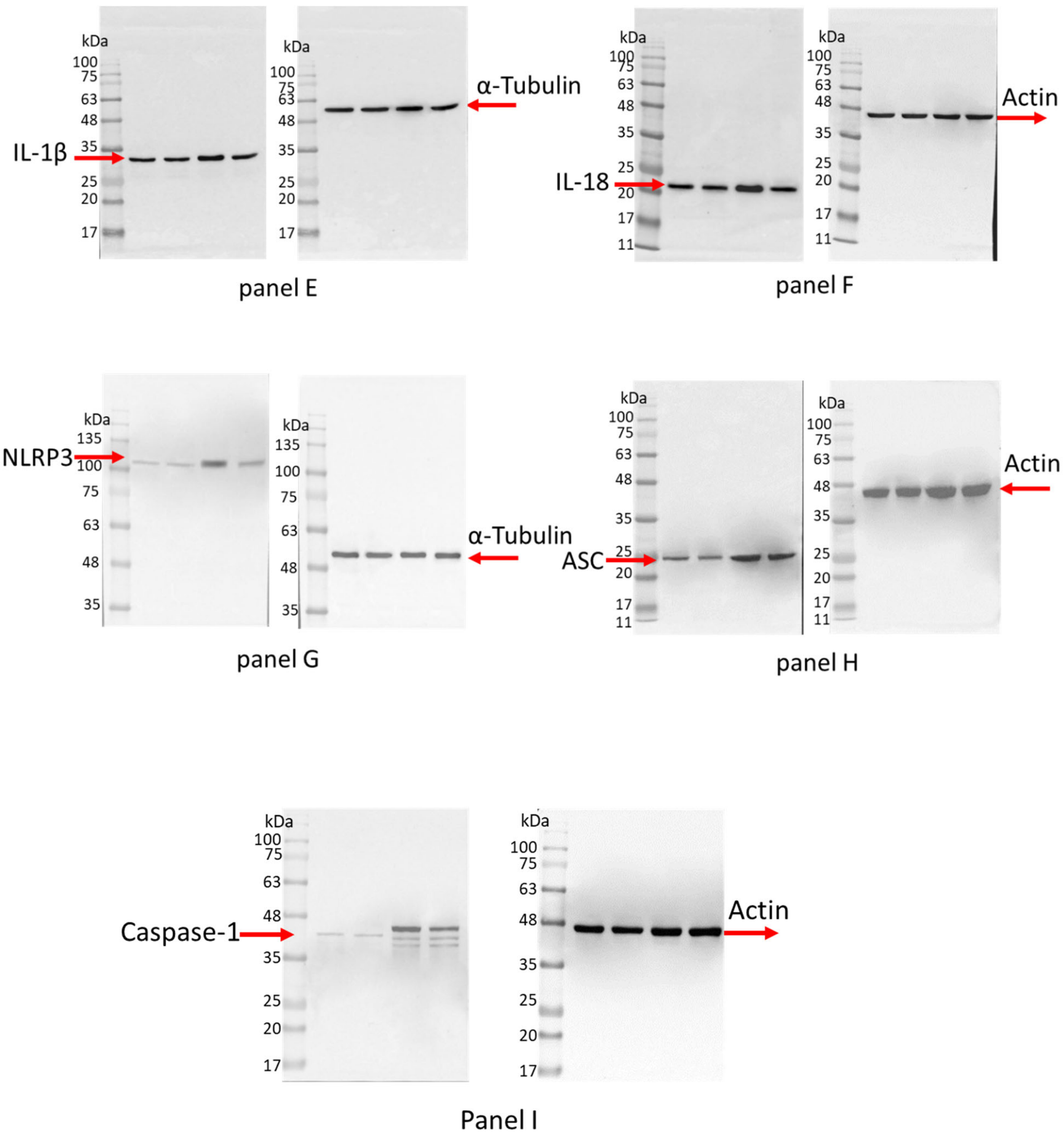

Figure S10: Image reporting untouched, uncropped gels related to Figure 5.

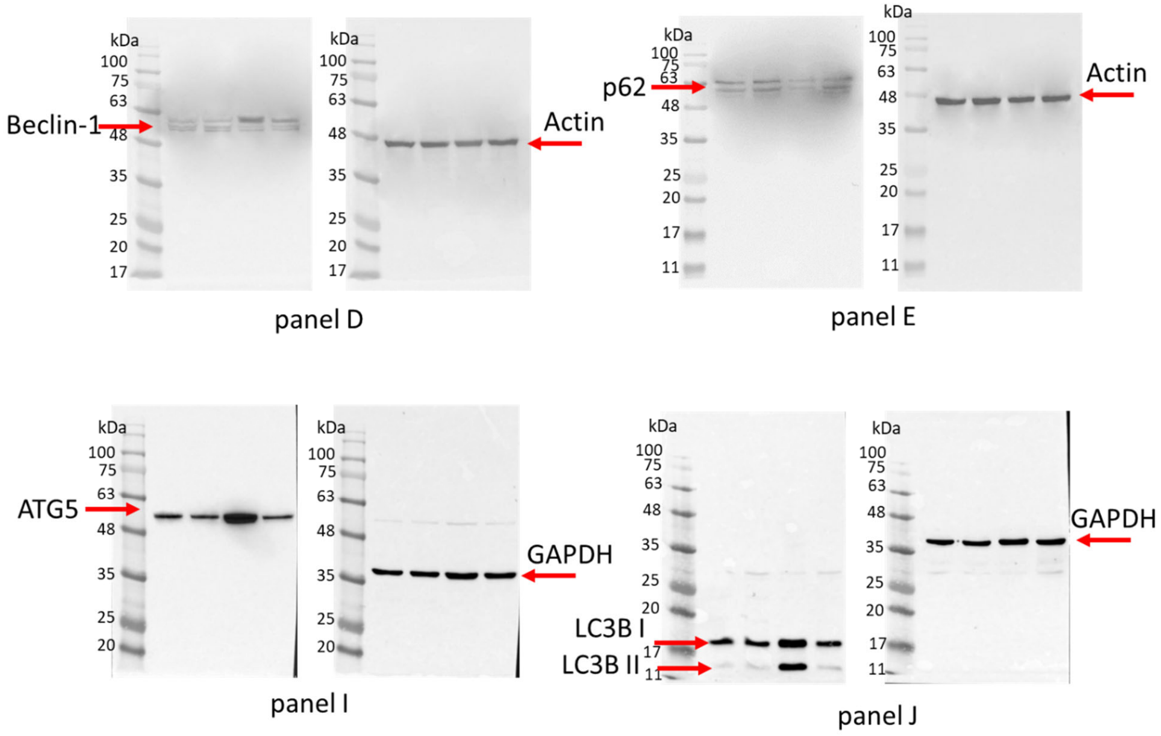

Figure S11: Image reporting untouched, uncropped gels related to Figure 6.

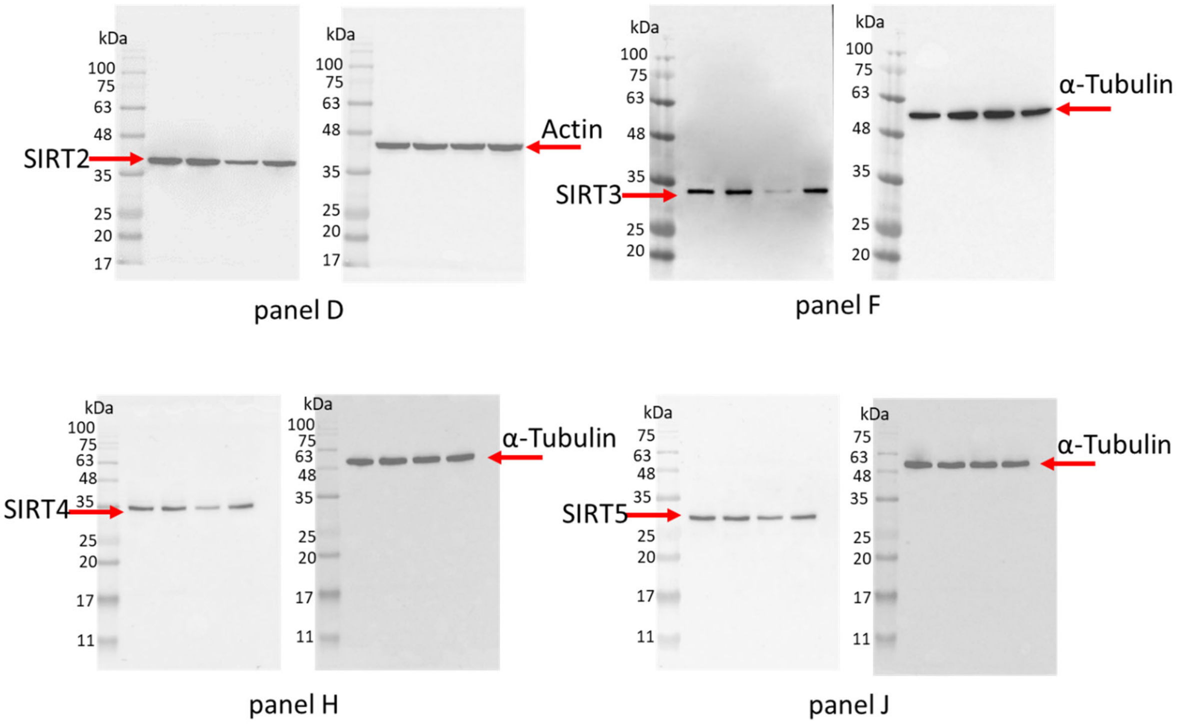

Figure S12: Image reporting untouched, uncropped gels related to Figure 7.

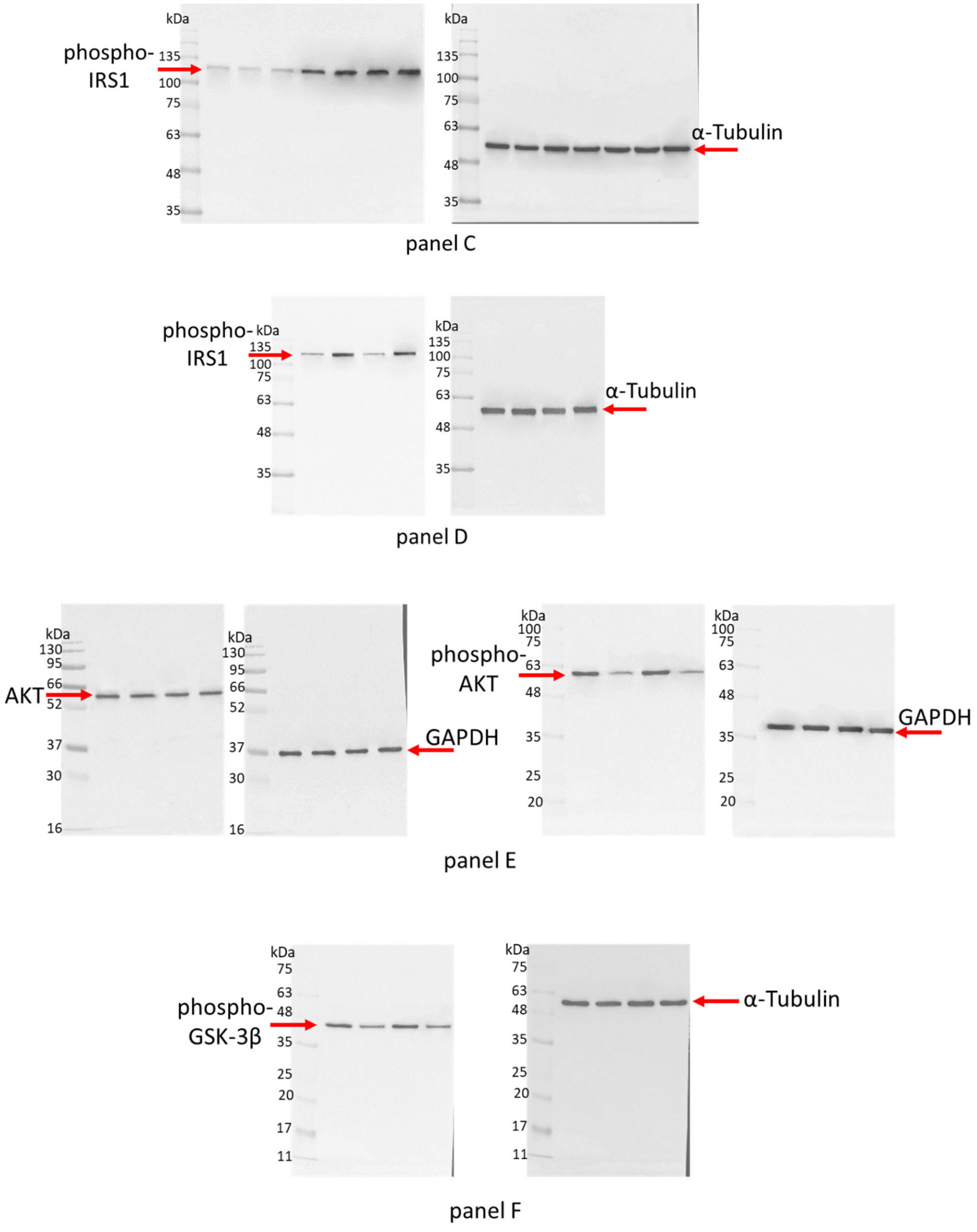

Figure S13: Image reporting untouched, uncropped gels related to Figure S1.

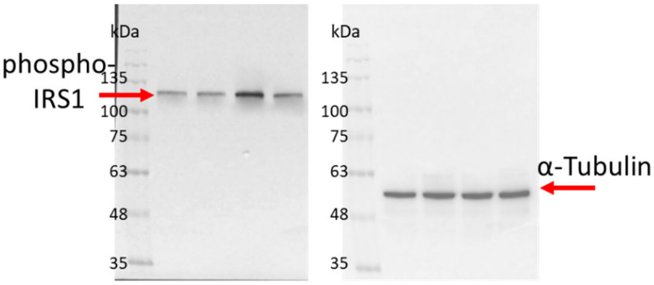

panel C

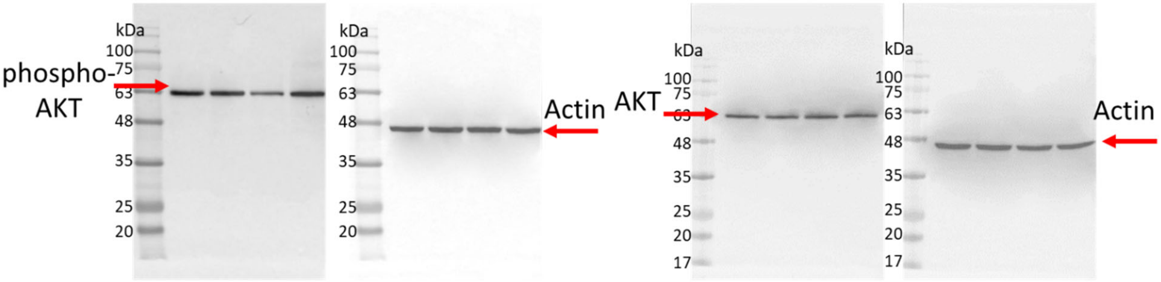

panel D

Figure S14: Image reporting untouched, uncropped gels related to Figure S5.
